# Association of CD33 genetic variants with neurocognitive profiles in chronic viral hepatitis

**DOI:** 10.1192/bjo.2025.10048

**Published:** 2025-07-10

**Authors:** Wei-Fang Tsai, Rwei-Ling Yu, Wan-Long Chuang, Jee-Fu Huang, Chia-Yen Dai, Yu-Wen Alvin Huang, Chun-Hsiang Tan

**Affiliations:** Graduate Institute of Clinical Medicine, College of Medicine, Kaohsiung Medical University, Kaohsiung, Taiwan; Institute of Behavioral Medicine, College of Medicine, National Cheng Kung University, Tainan, Taiwan; Hepatobiliary Division, Department of Internal Medicine and Hepatitis Center, Kaohsiung Medical University Hospital, Kaohsiung Medical University, Kaohsiung, Taiwan; Department of Molecular Biology, Cell Biology and Biochemistry, Brown University, Providence, Rhode Island, USA; Department of Neurology, Kaohsiung Medical University Hospital, Kaohsiung Medical University, Kaohsiung, Taiwan

**Keywords:** Chronic viral hepatitis, CD33, cognitive function, polymorphism, memory

## Abstract

**Background:**

CD33 has been implicated in the pathogenesis of Alzheimer’s disease primarily through its role in inhibiting the clearance of beta-amyloid (Aβ). However, genetic studies yield mixed results and it is unclear whether the impact of CD33 is specific to Alzheimer’s disease or related to broader neurodegenerative processes. Interestingly, CD33 has also been shown to interact with the hepatitis B (HBV) and C viruses (HCV).

**Aims:**

This study aims to investigate the effects of CD33 single-nucleotide polymorphisms (SNPs) on cognitive functions across diverse populations, including healthy controls, individuals with chronic HBV or HCV and those diagnosed with Parkinson’s disease.

**Method:**

We genotyped CD33 SNPs in 563 participants using the Affymetrix platform. Participants’ cognitive functions were cross-sectionally assessed using a neuropsychological test battery spanning six domains.

**Results:**

Our analysis revealed that CD33 SNP variations had no significant cognitive impact on healthy individuals or Parkinson’s disease patients. However, chronic HBV and HCV patients exhibited significant cognitive differences, particularly in memory, related to CD33 SNP genotypes. Moderation analysis indicated a heightened influence of CD33 SNPs on cognitive functions in chronic HBV and HCV individuals. Our data also suggest that inflammation severity may modulate the cognitive effects in hepatitis patients with specific CD33 SNPs.

**Conclusions:**

This study highlights the importance of CD33 SNPs in cognitive outcomes, emphasising their role in the context of chronic viral hepatitis. It contributes to understanding the cognitive profiles influenced by CD33 SNPs and posits CD33’s potential contribution to neurodegenerative disease progression, potentially intensified by HBV/HCV-induced inflammation.

CD33 is a transmembrane receptor principally expressed on both haematopoietic and phagocytic cells, including monocytes, dendritic cells and microglial cells, suggesting its significant role in immune responses.^1^ Although the precise role of CD33 in human physiology remains elusive, it has been shown in several studies to be involved in the pathogenesis of Alzheimer’s disease.

## CD33 in Alzheimer’s disease

The involvement of CD33 in Alzheimer’s disease is evidenced by the association between CD33 expression and disease status and dementia severity. Research indicates a link between CD33 overexpression in microglia and reduced beta-amyloid (Aβ) clearance, with APPSwe/PS1rE9 mouse studies showing that CD33 knockout decreases Aβ42 levels and plaque burden.^2^ This suggests that CD33 overexpression hinders microglial Aβ clearance in Alzheimer’s disease. Furthermore, CD33 deletion in a mouse model of Alzheimer’s disease was found to improve memory retention,^3^ underscoring CD33’s potential contribution to development of the disease.^4^

## CD33, viral hepatitis and cognitive impairment

Numerous genome-wide association studies have highlighted CD33 as a genetic risk factor for Alzheimer’s disease,^5,6^ although some studies report conflicting findings,^7,8^ indicating potential moderating factors influencing CD33’s role. Several studies provide evidence of a possible interaction between CD33 and viral hepatitis: specifically, hepatitis B virus (HBV) activation of CD33 on myeloid cells may facilitate immune evasion,^9^ and hepatitis C virus (HCV) increases CD33^+^ myeloid-derived suppressor cells (MDSCs), impairing T-cell function.^10,11^ This indicates a complex relationship between CD33, viral hepatitis and immune regulation. Nevertheless, the link between viral hepatitis-induced CD33 activation and cognitive impairment is poorly understood. Additionally, it remains uncertain whether CD33’s association with neurodegenerative diseases extends beyond Alzheimer’s disease, which cognitive domains are most affected and the underlying mechanisms. Our study investigates the effect of CD33 SNPs on cognitive functions across populations, including those with chronic hepatitis B, chronic hepatitis C, Parkinson’s disease and healthy controls, to elucidate these relationships.

## Method

### Participants

This study enrolled 563 individuals >50 years of age, including 238 healthy controls, 61 with HBV, 93 with HCV and 171 with Parkinson’s disease. HBV diagnosis was confirmed by positive hepatitis B surface antigen, and HCV by positive anti-HCV immunoglobulin G.^12^ Data on demographics and medical history (age, gender, education level, fibrosis-4 (FIB-4) index,^13^ hepatitis status) were collected from interviews and medical records. The study excluded participants with <6 years of education or histories of untreated malignancy, substance abuse, psychiatric disorders requiring medical treatment, neurovascular disorders, end-stage renal disease, acute infectious conditions or co-infection with HBV or HCV. The exclusion of psychiatric disorders and untreated malignancy was utilised to avoid confounders in evaluating neurocognitive functions, ensuring a clearer focus on the effects of chronic viral hepatitis and CD33 single-nucleotide polymorphism (SNP) variations.

All procedures contributing to this work comply with the ethical standards of the relevant national and institutional committees on human experimentation, and with the Helsinki Declaration of 1975 as revised in 2013. All procedures involving human subjects were approved by the committee of Kaohsiung Medical University Hospital (approval nos. KMUHIRB-G(II)-20160001 and 20170022KMUHIRB-G(I)-20170022), and by that of National Cheng Kung University Hospital (approval no. --/B-ER-104-082), and all methods were performed following the approved guidelines. Written informed consent was obtained from all participants before their participation.

### Genotyping

Blood leukocytes were collected for genomic DNA extraction using the Geneaid kit. CD33 SNP genotyping was conducted using the Affymetrix GeneChip on the Axiom Genome-Wide TWB 2.0 Array Plate, specifically designed for the Taiwanese population. SNPs were selected to ensure relevant and applicable genotyping markers for the Taiwanese population and clinical applications. This array contains approximately 686 000 SNPs and, in our study, we analysed SNPs in the CD33 region spanning from the 2 kb upstream variant rs12985029 to rs111722079 at position 51235676. Results were checked for Hardy–Weinberg equilibrium (Supplementary Table 1). To increase statistical analysis power, minor allele carriers (homozygotes and heterozygotes) were combined. Analyses were conducted based on the three genotype groups of the selected SNPs to explore the dose–response effect of CD33 SNPs. With sample sizes of 61 for HBV and 93 for HCV and an expected effect size of 0.25, our analysis indicated that the power required to detect an effect at *α* = 0.05 was 38.0 and 55.5%, respectively. This suggests that the small sample sizes of participants carrying the homozygous minor allele in the viral hepatitis groups limited our ability to reject the null hypothesis correctly. However, results based on the three genotype groups are provided in Supplementary Tables 3(a), 4(a), 5(a), 6(a), 7 and 8(a) for transparency. Haplotype frequencies and linkage disequilibrium among SNPs were analysed using Haploview 4.2 software, employing the haplotype block method for block definition.^14^

### Cognitive measures

The study participants underwent a global cognitive screening with the Mini-Mental State Examination (MMSE), followed by a neuropsychological battery to assess various neurocognitive domains, including executive function, visuospatial function, memory, psychomotor speed, attention and language function. Neurocognitive assessment tools were employed similarly to our previous study,^15^ including Trial 2 of the Colour Trails Test (CTT) (executive function),^16^ the Pentagons Copy of MMSE (visuospatial function), selected subtests from the Wechsler Memory Scale-III (specifically, the Logical Memory and Visual Reproduction subtests to evaluate memory), Trial 1 of the CTT (psychomotor speed),^16^ Paced Auditory Serial Addition Test (PASAT, attention)^17^ and several components of MMSE for language function. We used the raw scores from these neuropsychological tests for further analysis.

### Statistical analysis

Statistical analyses were conducted using IBM SPSS Statistics for Windows, Version 22.0. Quantitative variables were presented as means ± standard deviations, while qualitative variables were reported as frequencies or percentages. Normality was assessed using the Kolmogorov–Smirnov test. The Mann–Whitney *U*-test was used to evaluate differences between two groups with non-normally distributed quantitative variables, while qualitative variables were tested among stratified groups using a chi-square test. Differences in demographic characteristics between study groups were assessed using the Kruskal–Wallis test. Regression analyses were conducted using the PROCESS macro in SPSS, adjusting for age, gender and education level as covariates, to investigate whether different diseases modulate the impact of CD33 polymorphism on cognitive function. Moderation analysis included various diseases as the moderator variable (*W*), neuropsychological test scores as outcome variables (*Y*) and CD33 SNPs as independent variables (*X*). To investigate the potential impact of inflammation on the association between CD33 SNPs and cognitive function in individuals with hepatitis, FIB-4 was utilised as the moderator variable (*W*). Subsequently, the interaction between CD33 and inflammation regarding cognitive function was examined. To address the issue of multiple comparisons, *P*-values were adjusted using the Benjamini–Hochberg procedure to control the false discovery rate (FDR). An FDR-adjusted *P*-value (or *q*-value) <0.05 indicates statistical significance.^18^ In our study, data points with missing values were excluded from the analysis, as detailed in Tables 1 and 4.

**Table 1:** Effects of CD33 SNPs on the cognitive functions of participants in the healthy control group

SNP	rs12985029 (*n* = 235)			rs3826656 (*n* = 237)			rs33978622 (*n* = 237)		
Genotype	AA + AG (*n* = 87)	GG (*n* = 148)	Statistic	AA + AG (*n* = 112)	GG (*n* = 125)	Statistic	CC + CG (*n* = 78)	GG (*n* = 159)	Statistic
Age (years)	63.92 ± 7.449	64.82 ± 6.015	*P* = 0.412	64.04 ± 7.082	65.07 ± 6.221	*p* = 0.218	63.62 ± 7.451	65.06 ± 6.186	*p* = 0.145
Male/female	18/69	45/103	*P* = 0.105	27/85	37/88	*p* = 0.343	18/60	46/113	*p* = 0.341
Education (years)	12.63 ± 3.612	12.88 ± 3.391	*P* = 0.705	12.97 ± 3.612	12.62 ± 3.314	*p* = 0.453	12.87 ± 3.241	12.74 ± 3.564	*p* = 0.927
Global cognitive screening									
MMSE	27.29 ± 2.005	27.53 ± 2.254	*P* = 0.220 *q* = 0.880	27.34 ± 1.843	27.54 ± 2.408	*p* = 0.110 *q* = 0.804	27.37 ± 1.596	27.48 ± 2.389	*p* = 0.113 *q* = 0.804
Executive function									
Colour Trails Test 2	108.11 ± 44.329	110.13 ± 42.926	*P* = 0.567 *q* = 0.880	108.16 ± 45.685	110.76 ± 41.322	*p* = 0.296 *q* = 0.880	103.01 ± 41.321	112.73 ± 44.108	*p* = 0.057 *q* = 0.804
Visuospatial function									
Pentagons Copy (MMSE)	0.87 ± 0.334	0.91 ± 0.284	*P* = 0.346 *q* = 0.880	0.88 ± 0.322	0.91 ± 0.284	*p* = 0.475 *q* = 0.880	0.91 ± 0.288	0.89 ± 0.310	*p* = 0.681 *q* = 0.963
Memory									
Wechsler Memory Scale-III									
Logical memory									
Immediate	33.92 ± 11.175	35.05 ± 10.900	*P* = 0.443 *q* = 0.880	34.41 ± 10.689	34.74 ± 11.240	*p* = 0.741 *q* = 0.963	34.55 ± 9.328	34.60 ± 11.707	*p* = 0.738 *q* = 0.963
Delayed	20.98 ± 9.366	21.74 ± 8.312	*P* = 0.540 *q* = 0.880	21.26 ± 8.929	21.57 ± 8.484	*p* = 0.811 *q* = 0.968	21.58 ± 8.495	21.35 ± 8.794	*p* = 0.967 *q* = 0.968
Visual reproduction									
Immediate	75.38 ± 15.709^a^	73.75 ± 14.324^b^	*P* = 0.243 *q* = 0.880	75.39 ± 15.330^a^	73.33 ± 14.354^b^	*p* = 0.134 *q* = 0.804	74.99 ± 13.853^b^	73.97 ± 15.300^a^	*p* = 0.554 *q* = 0.880
Delayed	51.20 ± 19.390^a^	51.88 ± 21.894^b^	*P* = 0.897 *q* = 0.968	51.33 ± 19.854^a^	52.09 ± 21.991^b^	*p* = 0.893 *q* = 0.968	52.54 ± 19.897^b^	51.34 ± 21.523^a^	*p* = 0.531 *q* = 0.880
Psychomotor speed									
Colour Trails Test 1	50.27 ± 18.043	53.22 ± 23.987	*P* = 0.555 *q* = 0.880	50.75 ± 18.767	53.42 ± 24.365	*p* = 0.500 *q* = 0.880	48.67 ± 17.500	53.87 ± 23.617	*p* = 0.113 *q* = 0.804
Attention									
Paced Auditory Serial Addition Test	71.69 ± 18.231	73.23 ± 17.416	*P* = 0.587 *q* = 0.880	74.08 ± 17.667	71.50 ± 17.671	*p* = 0.230 *q* = 0.880	72.47 ± 17.599	72.84 ± 17.772	*p* = 0.770 *q* = 0.963
Language									
Language (MMSE)	4.84 ± 0.428	4.85 ± 0.393	*P* = 0.931 *q* = 0.968	4.85 ± 0.407	4.85 ± 0.403	*p* = 0.968 *q* = 0.968	4.87 ± 0.373	4.84 ± 0.419	*p* = 0.532 *q* = 0.880

SNP, single-nucleotide polymorphism; MMSE, Mini-Mental state examination. The *q*-value used here is false discovery rate, employed to correct the results of multiple comparisons involving three SNPs.

a Five missing values.

b Four missing values.

## Results

### Demographic characteristics and neuropsychological test scores by study group

Participants’ demographic characteristics and neuropsychological test scores, including 238 healthy controls, 61 with HBV, 93 with HCV and 171 with PD, are detailed in Supplementary Table 2. Among participants with HBV, 36 were receiving antiviral treatment while 67 of the HCV-positive participants had achieved sustained virologic response. The mean aspartate aminotransferase (AST) and alanine aminotransferase (ALT) levels in the viral hepatitis groups were below twice the upper limit of normal, indicating that most participants were not experiencing acute exacerbations. Significant differences were observed between the groups in terms of age (*P* < 0.001), gender distribution (*P* < 0.001) and years of education (*P* < 0.001). These variables were included as covariates in the moderation analysis, to minimise potential confounding effects when analysing all participants together.

Regarding global cognitive function, assessed using MMSE, individuals with Parkinson’s disease showed significantly worse performance than those in the HBV group (*P* < 0.001). When assessing domain-specific cognitive functions, significant differences were observed across executive function, memory, psychomotor speed and attention. For instance, individuals with HCV had significantly lower scores on memory tasks compared with healthy controls, while psychomotor speed and executive function, as measured by Colour Trails Test 1 and 2, respectively, were significantly slower in Parkinson’s disease individuals compared with several of the other groups. Furthermore, individuals with Parkinson’s disease also showed significantly worse performance on memory tasks and attention compared with healthy controls.

### Analysis of genotype and allele frequencies in the CD33 gene region

We analysed 11 SNPs in the CD33 gene region, focusing on those with minor allele frequency (MAF) >10%, namely rs12985029, rs3826656, rs3865444, rs12459419 and rs33978622. Allele frequencies of these SNPs in our study (Supplementary Table 1 and Supplementary Fig. 1) were similar to the results from the East Asia data-set in the Allele Frequency Aggregator project.^19^ Of note, there is a difference in the allele frequency of rs3826656 between the East Asian and global populations. In the latter, allele A had a frequency of 0.7452 while in the former it was 0.2950, highlighting varying allele distributions among different ethnicities. To further enhance statistical power and group sizes, we prioritised SNPs with the highest MAF in blocks showing strong linkage disequilibrium (Supplementary Table 1 and Supplementary Fig. 1). Consequently, we selected rs3826656 as the representative SNP within the block containing rs3826656, rs3865444 and rs12459419, with the two other SNPs included in Supplementary materials.

### Effect of CD33 SNPs on cognitive functions in the healthy control group

We combined minor allele homozygous and heterozygous individuals and compared their cognitive functions with those of major allele homozygous individuals. Among the healthy control group, we found no significant difference in cognitive functions between individuals with different genotype groups of the evaluated CD33 SNPs (Table 1 and Supplementary Table 3(a) and (b)), suggesting that the evaluated CD33 SNPs (rs12985029, rs3826656, rs3865444, rs12459419 and rs33978622) did not have a substantial impact on cognitive functions in the healthy control group recruited in the present study.

### The significant impact of CD33 SNPs on individuals with chronic HBV and HCV infections

In individuals with chronic HBV infection, several CD33 SNPs revealed a significant impact on cognitive performance. Specifically, individuals with the GG genotype of rs12985029 and rs33978622 performed significantly worse on MMSE scores (*q* = 0.044 for both), with average scores for those with the A allele (AA or AG genotype) of rs12985029 at 28.71 ± 1.102 and for the GG genotype at 27.85 ± 1.292 (Table 2). A similar pattern emerged for rs33978622, with mean scores at 28.74 ± 1.147 for C allele carriers (CC or CG genotype) and 27.88 ± 1.273 for GG genotype carriers (Table 2). Similarly, those with the CC genotype of rs3865444 and rs12459419 also yielded significantly lower MMSE scores (*q* = 0.039) (Supplementary Table 4(b)).

**Table 2:** Effects of CD33 SNPs on the cognitive functions of participants with chronic viral hepatitis B

SNP	rs12985029 (*n* = 61)			rs3826656 (*n* = 61)			rs33978622 (*n* = 61)		
Genotype	AA + AG (*n* = 21)	GG (*n* = 40)	Statistic	AA + AG (*n* = 26)	GG (*n* = 35)	Statistic	CC + CG (*n* = 19)	GG (*n* = 42)	Statistic
Age (years)	58.48 ± 4.750	61.08 ± 6.859	*P* = 0.169	59.31 ± 5.577	60.83 ± 6.789	*p* = 0.376	58.47 ± 4.846	60.95 ± 6.764	*p* = 0.201
Male/female	15/6	29/11	*P* = 0.930	19/7	25/10	*p* = 0.888	15/4	29/13	*p* = 0.428
Education (years)	14.29 ± 2.572	12.75 ± 3.418	*P* = 0.095	13.69 ± 2.950	12.97 ± 3.408	*p* = 0.432	15.00 ± 2.646	12.50 ± 3.172	*p* = 0.004*
Global cognitive screening									
MMSE	28.71 ± 1.102	27.85 ± 1.292	*P* = 0.015 *q* = 0.044*	28.42 ± 1.270	27.94 ± 1.282	*p* = 0.158 *q* = 0.192	28.74 ± 1.147	27.88 ± 1.273	*p* = 0.018 *q* = 0.044*
Executive function									
Colour Trails Test 2	87.50 ± 31.052	107.23 ± 43.890	*P* = 0.054 *q* = 0.085	85.53 ± 28.597	111.52 ± 45.133	*p* = 0.010 *q* = 0.038*	83.96 ± 29.769	107.89 ± 43.148	*p* = 0.018 *q* = 0.044*
Visuospatial function									
Pentagons Copy (MMSE)	0.95 ± 0.218	0.85 ± 0.362	*P* = 0.237 *q* = 0.245	0.96 ± 0.196	0.83 ± 0.382	*p* = 0.110 *q* = 0.143	1.00 ± 0.000	0.83 ± 0.377	*p* = 0.061 *q* = 0.092
Memory									
Wechsler Memory Scale-III									
Logical memory									
Immediate	39.48 ± 9.668	29.73 ± 8.608	*P* = 0.001 *q* = 0.010*	37.69 ± 10.575	29.66 ± 8.242	*p* = 0.002 *q* = 0.010*	39.42 ± 9.777	30.21 ± 8.888	*p* = 0.002 *q* = 0.010*
Delayed	25.10 ± 8.068	17.20 ± 6.992	*P* = 0.001 *q* = 0.010*	22.92 ± 9.029	17.69 ± 6.902	*p* = 0.019 *q* = 0.044*	24.79 ± 8.148	17.71 ± 7.353	*p* = 0.003 *q* = 0.013*
Visual reproduction									
Immediate	79.81 ± 10.787	73.18 ± 12.794	*P* = 0.049 *q* = 0.085	79.12 ± 10.386	72.74 ± 13.300	*p* = 0.046 *q* = 0.085	80.16 ± 10.890	73.33 ± 12.662	*p* = 0.051 *q* = 0.085
Delayed	67.00 ± 17.632	48.00 ± 17.355	*P* = 0.001 *q* = 0.010*	61.81 ± 19.610	49.14 ± 17.928	*p* = 0.025 *q* = 0.054	66.26 ± 17.935	49.24 ± 18.047	*p* = 0.002 *q* = 0.010*
Psychomotor speed									
Colour Trails Test 1	41.21 ± 11.692	49.94 ± 26.574	*P* = 0.230 *q* = 0.245	41.04 ± 10.880	51.32 ± 28.069	*p* = 0.215 *q* = 0.239	40.10 ± 11.769	50.03 ± 25.917	*p* = 0.090 *q* = 0.123
Attention									
Paced Auditory Serial Addition Test	81.43 ± 10.117	73.00 ± 12.890	*P* = 0.016 *q* = 0.044*	78.73 ± 10.562	73.80 ± 13.670	*p* = 0.186 *q* = 0.215	80.47 ± 12.140	73.83 ± 12.372	*p* = 0.050 *q* = 0.085
Language									
Language (MMSE)	4.86 ± 0.359	4.65 ± 0.483	*P* = 0.089 *q* = 0.123	4.73 ± 0.452	4.71 ± 0.458	*p* = 0.888 *q* = 0.888	4.84 ± 0.375	4.67 ± 0.477	*p* = 0.160 *q* = 0.192

SNP, single-nucleotide polymorphism; MMSE, Mini-Mental State Examination. The *q*-value used here is false discovery rate, employed to correct the results of multiple comparisons involving three SNPs. Bold: *q < 0.05.

A similar effect of CD33 SNP was also found in Trial 2 of CTT scores. GG genotype carriers of rs3826656 and rs33978622 yielded significantly lower scores (*q* = 0.038 and 0.044, respectively) than A and C allele carriers (Table 2). This pattern was also observed for individuals with the CC genotype of rs3865444 and rs12459419 (*q* = 0.039) (Supplementary Table 4(b)). In addition, among the HBV group, individuals carrying the GG genotype of rs12985029, rs3826656 and rs33978622 exhibited significantly poorer performance in both immediate and delayed recall of logical memory. For immediate recall of logical memory, mean scores were 39.48 ± 9.668 for those carrying the A allele (AA or AG genotypes), while those with the GG genotype of rs12985029 had mean scores of 29.73 ± 8.608 (*q* = 0.010). Moreover, participants carrying the A allele (AA or AG genotypes) had mean scores of 37.69 ± 10.575, as opposed to 29.66 ± 8.242 for those with the GG genotype of rs3826656 (*q* = 0.010). Showing a similar pattern, those carrying the C allele (CC or CG genotypes) had mean scores of 39.42 ± 9.777 while those with the GG genotype of rs33978622 yielded 30.21 ± 8.888 (*q* = 0.010) (Table 2). It is also noteworthy that individuals with the CC genotype of rs3865444 and rs12459419 performed significantly worse on immediate recall of logical memory (*q* = 0.010) (Supplementary Table 4(b)). For delayed recall of logical memory, mean scores were 25.10 ± 8.068 for carriers of the A allele (genotypes AA or AG) and 17.20 ± 6.992 for those with the GG genotype of rs12985029 (*q* = 0.010). Similarly, mean scores were 22.92 ± 9.029 for carriers of the A allele (genotypes AA or AG) and 17.69 ± 6.902 for those with the GG genotype of rs3826656 (*q* = 0.044). Similarly, carriers of the C allele (CC or CG genotypes) had mean scores of 24.79 ± 8.148 while those with the GG genotype of rs33978622 averaged 17.71 ± 7.353 (*q* = 0.013) (Table 2). Furthermore, individuals with the CC genotypes of rs3865444 and rs12459419 also scored significantly lower in delayed recall of logical memory (*q* = 0.012) (Supplementary Table 4(b)).

Furthermore, individuals possessing the GG genotypes of rs12985029 and rs33978622 demonstrated significantly inferior performance on the delayed recall of visual reproduction (Table 2), another measure of memory function. For those carrying the A allele (AA or AG genotypes), mean scores averaged 67.00 ± 17.632 compared with 48.00 ± 17.355 for those with the GG genotype of rs12985029 (*q* = 0.010). Likewise, tmean scores were 66.26 ± 17.935 for participants carrying the C allele (CC or CG genotypes) versus 49.24 ± 18.047 for those with the GG genotype of rs33978622 (*q* = 0.010) (Table 2). Similarly, individuals with the CC genotypes of rs3865444 and rs12459419 also scored significantly lower on delayed recall of visual reproduction (*q* = 0.010) (Supplementary Table 4(b)).

Moreover, individuals with the GG genotype of rs12985029 scored significantly lower on PASAT, indicative of attention function impairment, in which mean scores were 81.43 ± 10.117 for carriers of the A allele (AA or AG genotypes), contrasting with 73.00 ± 12.890 for those with the GG genotype of rs12985029 (*q* = 0.044) (Table 2).

Similar to the observation among individuals with HBV, a significant effect of CD33 SNPs on cognitive functions was noted within the HCV cohort (Table 3 and Supplementary Table 5(b)). Individuals with the GG genotype of rs3826656 performed significantly worse in delayed recall of logical memory. Mean scores for carriers of the A allele (genotypes AA or AG) were 18.35 ± 7.433, while those with the GG genotype of rs3826656 had a mean score of 13.40 ± 6.737 (*q* = 0.030) (Table 3).

**Table 3:** Effects of CD33 SNPs on the cognitive functions of participants with chronic viral hepatitis C

SNP	rs12985029 (*n* = 93)			rs3826656 (*n* = 93)			rs33978622 (*n* = 93)		
Genotype	AA + AG (*n* = 43)	GG (*n* = 50)	Statistic	AA + AG (*n* = 48)	GG (*n* = 45)	Statistic	CC + CG (*n* = 41)	GG (*n* = 52)	Statistic
Age (years)	62.56 ± 7.863	62.92 ± 7.597	*P* = 0.844	63.15 ± 7.803	62.33 ± 7.613	*p* = 0.606	63.24 ± 7.426	62.37 ± 7.926	*p* = 0.567
Male/female	15/28	18/32	*P* = 0.911	14/34	19/26	*p* = 0.191	15/26	18/34	*p* = 0.845
Education (years)	9.88 ± 3.389	11.36 ± 3.735	*P* = 0.059	10.19 ± 3.233	11.20 ± 3.992	*p* = 0.264	10.51 ± 3.487	10.81 ± 3.778	*p* = 0.806
Global cognitive screening									
MMSE	27.30 ± 1.807	27.24 ± 2.387	*P* = 0.541 *q* = 0.955	27.33 ± 2.025	27.20 ± 2.252	*p* = 0.972 *q* = 0.976	27.27 ± 1.817	27.27 ± 2.361	*p* = 0.433 *q* = 0.874
Executive function									
Colour Trails Test 2	119.70 ± 41.171	128.58 ± 68.769	*P* = 0.865 *q* = 0.976	120.99 ± 50.438	128.19 ± 64.661	*p* = 0.706 *q* = 0.976	111.89 ± 39.810	134.39 ± 67.117	*p* = 0.144 q = 0.813
Visuospatial function									
Pentagons Copy (MMSE)	0.81 ± 0.394	0.84 ± 0.370	*P* = 0.741 *q* = 0.976	0.81 ± 0.394	0.84 ± 0.367	*p* = 0.685 *q* = 0.976	0.83 ± 0.381	0.83 ± 0.382	*p* = 0.976 *q* = 0.976
Memory									
Wechsler Memory Scale-III									
Logical memory									
Immediate	29.91 ± 10.636	26.02 ± 10.807	*P* = 0.047 *q* = 0.352	30.29 ± 11.026	25.18 ± 10.116	*p* = 0.015 *q* = 0.225	28.66 ± 10.523	27.15 ± 11.152	*p* = 0.298 *q* = 0.813
Delayed	17.47 ± 7.333	14.66 ± 7.455	*P* = 0.047 *q* = 0.352	18.35 ± 7.433	13.40 ± 6.737	*p* = 0.001 *q* = 0.030*	17.05 ± 7.379	15.10 ± 7.539	*p* = 0.169 *q* = 0.813
Visual reproduction									
Immediate	70.02 ± 13.068	65.22 ± 17.223	*P* = 0.225 *q* = 0.813	69.00 ± 14.546	65.78 ± 16.553	*p* = 0.430 *q* = 0.874	69.98 ± 12.605	65.44 ± 17.385	*p* = 0.207 *q* = 0.813
Delayed	45.09 ± 20.218	41.60 ± 24.619	*P* = 0.436 *q* = 0.874	44.17 ± 20.778	42.20 ± 24.665	*p* = 0.715 *q* = 0.976	43.90 ± 21.482	42.67 ± 23.703	*p* = 0.865 *q* = 0.976
Psychomotor speed									
Colour Trails Test 1	55.49 ± 28.827	52.54 ± 26.985	*P* = 0.254 *q* = 0.813	54.90 ± 28.428	52.84 ± 27.263	*p* = 0.437 *q* = 0.874	49.81 ± 16.918	57.13 ± 33.754	*p* = 0.526 *q* = 0.955
Attention									
Paced Auditory Serial Addition Test	65.28 ± 18.553	64.84 ± 22.265	*P* = 0.856 *q* = 0.976	65.23 ± 18.383	64.84 ± 22.796	*p* = 0.887 *q* = 0.976	67.32 ± 19.539	63.25 ± 21.283	*p* = 0.292 *q* = 0.813
Language									
Language (MMSE)	4.86 ± 0.413	4.90 ± 0.303	*P* = 0.774 *q* = 0.976	4.88 ± 0.393	4.89 ± 0.318	*p* = 0.943 *q* = 0.976	4.85 ± 0.422	4.90 ± 0.298	*p* = 0.665 *q* = 0.976

SNP, single-nucleotide polymorphism; MMSE, Mini-Mental State Examination. The *q*-value used here is false discovery rate, employed to correct the results of multiple comparisons involving three SNPs. Bold: *q < 0.05.

### Absence of significant impact of CD33 SNPs on cognitive functions in the Parkinson’s disease group

To examine whether the effect of CD33 SNPs on cognitive functions is specific to individuals with viral hepatitis or common to all neurodegenerative diseases, we recruited individuals diagnosed with Parkinson’s disease into the present study. Despite observing a significant influence of CD33 SNPs on individuals with viral hepatitis, our analysis did not identify any significant differences in cognitive functions among Parkinson’s disease participants with different genotypes (Table 4 and Supplementary Table 6(b)). This suggests that the five CD33 SNPs under investigation (rs12985029, rs3826656, rs3865444, rs12459419 and rs33978622) did not exert a significant impact on the cognitive functions of individuals diagnosed with Parkinson’s disease.

**Table 4:** Effects of CD33 SNPs on the cognitive functions of participants with Parkinson’s disease

SNP	rs12985029 (*n* = 171)			rs3826656 (*n* = 171)			rs33978622 (*n* = 170)		
Genotype	AA + AG (*n* = 65)	GG (*n* = 106)	Statistic	AA + AG (*n* = 81)	GG (*n* = 90)	Statistic	CC + CG (*n* = 69)	GG (*n* = 101)	Statistic
Age (years)	64.62 ± 6.964	66.37 ± 7.659	*P* = 0.175	66.33 ± 7.265	65.13 ± 7.572	*p* = 0.175	65.49 ± 6.878	65.82 ± 61.588	*p* = 0.869
Male/female	42/23	73/33	*P* = 0.566	54/27	61/29	*p* = 0.878	42/27	73/28	*p* = 0.120
Education (years)	12.15 ± 3.555	12.61 ± 3.671	*P* = 0.351	11.91 ± 3.655	12.91 ± 3.549	*p* = 0.058	11.79 ± 3.588	12.86 ± 3.617	*p* = 0.037
Global cognitive screening									
MMSE	27.12 ± 2.601	26.75 ± 3.005	*P* = 0.556 *q* = 0.877	26.75 ± 3.080	27.01 ± 2.650	*p* = 0.760 *q* = 0.932	26.70 ± 3.305	27.04 ± 2.522	*p* = 0.847 *q* = 0.941
Executive function									
Colour Trails Test 2	131.36 ± 68.287^b^	144.66 ± 79.899^c^	*P* = 0.257 *q* = 0.796	145.28 ± 85.806^j^	134.45 ± 65.281^k^	*p* = 0.432 *q* = 0.877	136.82 ± 59.471^n^	141.47 ± 85.525^f^	*p* = 0.564 *q* = 0.877
Visuospatial function									
Pentagons Copy (MMSE)	0.88 ± 0.331	0.84 ± 0.369	*P* = 0.504 *q* = 0.877	0.85 ± 0.357	0.86 ± 0.354	*p* = 0.946 *q* = 0.980	0.87 ± 0.339	0.84 ± 0.367	*p* = 0.614 *q* = 0.877
Memory									
Wechsler Memory Scale-III									
Logical memory									
Immediate	28.69 ± 13.303	29.60 ± 12.797	*P* = 0.531 *q* = 0.877	27.22 ± 12.548	31.09 ± 13.121	*p* = 0.037 *q* = 0.796	27.54 ± 13.094	30.36 ± 12.849	*p* = 0.107 *q* = 0.796
Delayed	16.25 ± 10.749	16.58 ± 9.810	*P* = 0.800 *q* = 0.932	15.06 ± 10.037	17.70 ± 10.138	*p* = 0.076 *q* = 0.796	15.26 ± 10.657	17.23 ± 9.802	*p* = 0.205 *q* = 0.796
Visual reproduction									
Immediate	67.97 ± 18.576^f^	66.42 ± 18.894^g^	*P* = 0.491 *q* = 0.877	65.01 ± 19.346^f^	68.78 ± 18.095^g^	*p* = 0.269 *q* = 0.796	65.83 ± 18.333^o^	67.55 ± 19.028^k^	*p* = 0.604 *q* = 0.877
Delayed	45.25 ± 24.783^f^	40.64 ± 24.042^g^	*P* = 0.292 *q* = 0.796	39.27 ± 24.607^f^	45.13 ± 23.920^g^	*p* = 0.107 *q* = 0.796	39.00 ± 24.544^o^	44.30 ± 24.101^k^	*p* = 0.179 *q* = 0.796
Psychomotor speed									
Colour Trails Test 1	64.49 ± 37.320^q^	74.70 ± 49.860^n^	*P* = 0.176 *q* = 0.796	72.41 ± 48.691^a^	69.41 ± 42.953^f^	*p* = 0.682 *q* = 0.930	67.13 ± 33.962^n^	66.07 ± 32.236^f^	*p* = 0.726 *q* = 0.932
Attention									
Paced Auditory Serial Addition Test	64.65 ± 17.880^h^	65.71 ± 18.672^i^	*P* = 0.808 *q* = 0.932	63.19 ± 18.174^l^	67.47 ± 18.371^m^	*p* = 0.292 *q* = 0.796	65.23 ± 18.163^h^	65.68 ± 18.633^p^	*p* = 0.964 *q* = 0.980
Language									
Language (MMSE)	4.89 ± 0.312	4.84 ± 0.461	*P* = 0.611 *q* = 0.877	4.86 ± 0.379	4.86 ± 0.439	*p* = 0.980 *q* = 0.980	4.86 ± 0.493	4.86 ± 0.347	*p* = 0.520 *q* = 0.877

SNP, single-nucleotide polymorphism; MMSE, Mini-Mental State Examination. The *q*-value used here is false discovery rate, employed to correct the results of multiple comparisons involving three SNPs.

a. 5 missing values. ^b^4 missing values. ^c^13 missing values. ^d^27 missing values. ^e^58 missing values. ^f^9 missing values. ^g^12 missing values. ^h^34 missing values. ^i^51 missing values. ^j^7 missing values. ^k^10 missing values. ^l^38 missing values. ^m^47 missing values. ^n^8 missing values. ^o^11 missing values. ^p^50 missing values. ^q^6 missing values.

### Enhancement of CD33 SNPs effects among individuals with chronic viral hepatitis, but not in normal ageing or Parkinson’s disease group

The findings above suggest a significant influence of CD33 SNPs on cognitive functions within the cohort of individuals afflicted by chronic viral hepatitis, an impact not observed in the healthy control group or individuals with Parkinson’s disease. To examine whether the effect of CD33 SNPs was enhanced among individuals with HBV and HCV, we performed moderation analysis to evaluate the interactions between the impact of these SNPs and disease status in influencing cognitive functions, while age, gender and educational level were integrated into our moderation analysis to mitigate any potential confounding influences.

For rs12985029, significant interactions were identified between it and disease status with cognitive functions in the domain of memory, specifically in immediate recall of logical memory (*q* = 0.003) and delayed recall of logical memory (q = 0.003) (Table 5), in which significant effects of CD33 SNPs on immediate recall of logical memory were observed specifically in the HBV (*q* = 0.025) and HCV (*q* = 0.023) cohorts, but not in the healthy control (*q* = 0.418) or Parkinson’s disease groups (*q* = 0.540). The significant effect of CD33 SNPs on delayed recall of logical memory was also exclusively observed within the HBV (*q* = 0.023) and HCV (*q* = 0.023) groups, reinforcing the substantial role of CD33 SNPs among viral hepatitis patients.

**Table 5:** Moderation between CD33 SNPs and disease entity on cognitive functions

SNP	rs12985029	rs3826656	rs33978622
	Gene–diagnosis interaction (disease-specific SNP effect)	Gene–diagnosis interaction (disease-specific SNP effect)	Gene–diagnosis interaction (disease-specific SNP effect)
Global cognitive screening			
MMSE	*P* = 0.411; *q* = 0.649	*P* = 0.488; *q* = 0.713	*P* = 0.858; *q* = 0.914
Executive function			
Colour Trails Test 2	*P* = 0.752; *q* = 0.894	*P* = 0.309; *q* = 0.643	*P* = 0.374; *q* = 0.649
Visuospatial function			
Pentagons Copy (MMSE)	*P* = 0.499; *q* = 0.713	*P* = 0.349; *q* = 0.649	*P* = 0.288; *q* = 0.643
Memory			
Wechsler Memory Scale-III			
Logical memory			
Immediate	*P* = 0.003; *q* = 0.003* (HC: *P* = 0.314; *q* = 0.418; PD: *P* = 0.540; *q* = 0.540; HBV: *P* = 0.013; *q* = 0.025*; HCV: *P* = 0.006; *q* = 0.023*)	*P* < 0.001; *q* < 0.001* (HC: *P* = 0.314; *q* = 0.314; PD: *P* = 0.166; *q* = 0.222; HBV: *P* = 0.011; *q* = 0.022*; HCV: *P* = 0.002; *q* = 0.008*)	*P* = 0.093; *q* = 0.309
Delayed	*P* = 0.004; *q* = 0.003* (HC: *P* = 0.319; *q* = 0.425; PD: *P* = 0.663; *q* = 0.663; HBV: *P* = 0.008; *q* = 0.023*; HCV: *P* = 0.012; *q* = 0.023*)	*P* < 0.001; *q* < 0.001* (HC: *P* = 0.267; *q* = 0.275; PD: *P* = 0.275; *q* = 0.275; HBV: *P* = 0.042; *q* = 0.083; HCV: *P* < 0.001; *q* = 0.001*)	*P* = 0.061; *q* = 0.270
Visual reproduction			
Immediate	*P* = 0.322; *q* = 0.643	*P* = 0.220; *q* = 0.599	*P* = 0.315; *q* = 0.643
Delayed	*P* = 0.060; *q* = 0.270	*P* = 0.072; *q* = 0.270	*P* = 0.117; *q* = 0.350
Psychomotor speed			
Colour Trails Test 1	*P* = 0.629; *q* = 0.787	*P* = 0.914; *q* = 0.914	*P* = 0.068; *q* = 0.270
Attention			
Paced Auditory Serial Addition Test	*P* = 0.588; *q* = 0.767	*P* = 0.804; *q* = 0.894	*P* = 0.396; *q* = 0.649
Language			
Language (MMSE)	*P* = 0.525; *q* = 0.716	*P* = 0.906; *q* = 0.914	*P* = 0.802; *q* = 0.894

SNP, single-nucleotide polymorphism; MMSE, Mini-Mental State Examination; HC, healthy control; PD, Parkinson’s disease; HBV, hepatitis B virus; HCV hepatitis C virus. The *q*-value used here is false discovery rate, employed to correct the results of multiple comparisons involving three SNPs. Bold: *q < 0.05.

Similarly, our moderation analysis also revealed a significant interaction between disease status and rs3826656 in affecting immediate recall of logical memory (*q* < 0.001) and delayed recall of logical recall (*q* < 0.001) (Table 5). CD33 SNPs were found to have significant effects on immediate recall of logical memory, specifically within the HBV (*q* = 0.022) and HCV (*q* = 0.008) cohorts, while no such effects were observed in the healthy control (*q* = 0.314) or Parkinson’s disease groups (*q* = 0.222). A significant effect of rs3826656 on delayed recall of logical memory was found solely within the HCV cohort (*q* = 0.001) in moderation analysis, providing additional evidence of the enhanced effect of CD33 SNPs on cognitive performance in the context of viral hepatitis.

On the contrary, for SNP rs33978622, our moderation analysis did not disclose any significant interactions between different disease statuses and rs33978622 in influencing participants’ cognitive functions. To summarise, our moderation analysis highlights the specific enhancement of the effect of CD33 SNPs, namely rs12985029 and rs3826656, on cognitive functions in the context of viral hepatitis.

### Dissecting the role of CD33 SNPs in cognitive function: inflammation as a key moderator

To elucidate the mechanisms underlying the cognitive function differences conferred by CD33 SNPs among individuals with viral hepatitis, we examined the interactions between these SNPs and inflammation severity. Using FIB-4 as an inflammation biomarker, our moderation analysis identified a significant interaction between FIB-4 and rs12985029 (*q* = 0.012; Table 6). This interaction, coupled with the direct effect of rs12985029 (*q* = 0.045; Table 6), significantly moderated performance in Trial 1 of CTT. Our results underscore the importance of the rs12985029 genotype, whose impact is modulated by inflammation severity. Notably, among individuals with lower FIB-4 levels, those with viral hepatitis homozygous for the GG genotype of rs12985029 exhibited significantly worse performance in Trial 1 of CTT (*q* = 0.012; Supplementary Fig. 2) than those carrying the A allele. This effect was absent in those with higher FIB-4 levels.

**Table 6:** Moderation between CD33 SNPs and FIB-4 on cognitive functions in individuals with chronic viral hepatitis

SNP	rs12985029		rs3826656		rs33978622	
Study group (HBV + HCV)	*n* = 154					
Age (years)	61.73 ± 7.254					
Male/female	77/77					
Education (years)	11.71 ± 3.692					
Moderation analysis	Gene effect	Gene–inflammatory interaction	Gene effect	Gene–inflammatory interaction	Gene effect	Gene–inflammatory interaction
Global cognitive screening						
Mini-Mental State Examination	*P* = 0.786 *q* = 0.873	*P* = 0.274 *q* = 0.980	*P* = 0.523 *q* = 0.729	*P* = 0.193 *q* = 0.868	*P* = 0.631 *q* = 0.759	*P* = 0.437 *q* = 0.972
Executive function						
Colour Trails Test 2	*P* = 0.292 *q* = 0.673	*P* = 0.757 *q* = 0.980	*P* = 0.081 *q* = 0.388	*P* = 0.437 *q* = 0.980	*P* = 0.516 *q* = 0.729	*P* = 0.476 *q* = 0.980
Visuospatial function						
Pentagons Copy (MMSE)	*P* = 0.651 *q* = 0.759	*P* = 0.828 *q* = 0.980	*P* = 0.857 *q* = 0.918	*P* = 0.868 *q* = 0.980	*P* = 0.486 *q* = 0.729	*P* = 0.732 *q* = 0.980
Memory						
Wechsler Memory Scale-III						
Logical memory						
Immediate	*P* = 0.104 *q* = 0.390	*P* = 0.754 *q* = 0.980	*P* = 0.154 *q* = 0.463	*P* = 0.511 *q* = 0.980	*P* = 0.262 *q* = 0.655	*P* = 0.915 *q* = 0.980
Delayed	*P* = 0.012 *q* = 0.090	*P* = 0.515 *q* = 0.980	*P* = 0.009 *q* = 0.090	*P* = 0.653 *q* = 0.980	*P*= 0.0339 *q* = 0.2030	*P* = 0.345 *q* = 0.980
Visual reproduction						
Immediate	*P* = 0.964 *q* = 0.981	*P* = 0.146 *q* = 0.980	*P* = 0.199 *q* = 0.542	*P* = 0.906 *q* = 0.980	*P* = 0.474 *q* = 0.729	*P* = 0.789 *q* = 0.980
Delayed	*P* = 0.140 *q* = 0.463	*P* = 0.980 *q* = 0.993	*P* = 0.091 *q* = 0.388	*P* = 0.478 *q* = 0.980	*P* = 0.531 *q* = 0.729	*P* = 0.812 *q* = 0.980
Psychomotor speed						
Colour Trails Test 1	*P* = 0.002 *q* = 0.045*	*P* < 0.001 *q* = 0.012*	*P* = 0.004 *q* = 0.058	*P* = 0.006 *q* = 0.086	*P* = 0.535 *q* = 0.729	*P* = 0.068 *q* = 0.677
Attention						
Paced Auditory Serial Addition Test	*P* = 0.657 *q* = 0.759	*P* = 0.816 *q* = 0.980	*P* = 0.451 *q* = 0.729	*P* = 0.853 *q* = 0.980	*P* = 0.531 *q* = 0.729	*P* = 0.993 *q* = 0.993
Language						
Language (MMSE)	*P* = 0.624 *q* = 0.759	*P* = 0.868 *q* = 0.980	*P* = 0.530 *q* = 0.729	*P* = 0.369 *q* = 0.980	*P* = 0.981 *q* = 0.981	*P* = 0.703 *q* = 0.980

SNP, single-nucleotide polymorphism; MMSE, Mini-Mental State Examination; HBV, hepatitis B virus; HCV, hepatitis C virus. The *q*-value used here is false discovery rate, employed to correct the results of multiple comparisons involving three SNPs. Bold: *q < 0.05.

Additionally, our examination of the interactions between FIB-4 and two other SNPs, rs3865444 and rs12459419, both strongly linked with rs3826656, supports these observations. In particular, our analysis highlights FIB-4 as a crucial moderator of the effects of rs3865444 (*q* = 0.027; Supplementary Table 8(b)) and rs12459419 (*q* = 0.015; Supplementary Table 8(b)). The direct influence of both rs3865444 (*q* = 0.049; Supplementary Table 8(b)) and rs12459419 (*q* = 0.038; Supplementary Table 8(b)) also significantly shaped the performance in Trial 1 of CTT.

### Discussion

The role of CD33 in the pathogenesis of Alzheimer’s disease has garnered increasing attention. This study examined the associations between CD33 SNPs and cognitive functions across diverse cohorts, encompassing healthy individuals and those affected by HBV, HCV and Parkinson’s disease. Our findings reveal distinct impacts of CD33 SNPs on the cognitive profiles of different participant groups. Notably, HBV-affected individuals carrying the GG genotype of rs12985029 and rs33978622, as well as those with the CC genotype of rs3865444 and rs12459419, demonstrated significantly worse cognitive scores, especially in the memory domain. Additionally, HCV-affected individuals carrying the GG genotype of rs3826656 exhibited significantly poorer verbal memory. In contrast, no significant effect of CD33 SNPs on cognitive functions was identified among healthy controls or Parkinson’s disease-affected individuals. Further moderation analysis found disease-specific enhancement of the effect of CD33 SNPs, particularly rs12985029 and rs3826656, by HBV and HCV status influencing memory function, with the most pronounced effect on immediate and delayed verbal memory. The study also probed the interactions between inflammation severity (as assessed by the FIB-4 index) and the impact of CD33 SNPs on cognitive functions. We found that the substantial influence of rs12985029, rs3865444 and rs12459419 on cognitive functions, especially in Trial 1 of the CTT, was moderated by inflammation severity. This modulation was particularly pronounced among individuals with less severe inflammation. These results suggest an association between CD33 and cognitive functions, with the effect potentially being enhanced in the presence of chronic viral hepatitis. Furthermore, the systemic inflammation induced by chronic viral hepatitis moderates the CD33 SNP effect.

Several studies have probed the associations between CD33 and Alzheimer’s disease susceptibility, yet the precise role of CD33 in the pathogenesis of this condition remains undetermined. While one meta-analysis using data from studies of East Asian populations found a significant association between rs3826656 and the risk of Alzheimer’s disease (odds ratio 1.39, 95% CI 1.09–1.76),^20^ another employing three Asian cohorts and one Caucasian found no such association (odds ratio 0.94, 95% CI 0.62–1.41).^21^ Additionally, two recent large-scale studies yielded inconsistent findings regarding the relationship between CD33 and Alzheimer’s disease. One genome-wide association study comprising 1 126 563 participants found CD33 to be one of 38 genomic risk loci.^6^ In contrast, a study of 788 989 participants pinpointed 75 risk loci but notably excluded CD33 from its list of those identified.^8^ Such disparities underscore the complexity and heterogeneity inherent in Alzheimer’s disease and reaffirm the ambiguous role of CD33 in its pathogenesis. Our research showed a significant influence of CD33 SNPs on cognitive functions in individuals with chronic viral hepatitis, yet this effect was absent in healthy control and Parkinson’s disease cohorts. The significant effect of CD33 SNPs in those with chronic viral hepatitis might illuminate the varied findings from earlier studies, underscoring the potential impact of chronic viral hepatitis on CD33’s role in Alzheimer’s disease pathogenesis.

While CD33 has been posited as playing an important role in the pathogenesis of Alzheimer’s disease, which is frequently characterised by memory-related issues^22^ linked to medial temporal atrophy in the amygdala and hippocampus,^23^ investigations into the diverse cognitive profiles influenced by CD33 SNPs remain limited. Besides examining the potential effect of CD33 SNPs on cognition and identifying the population vulnerable to the effect of these SNPs, this study employed a battery of neuropsychological tests to investigate the diverse cognitive domains that may be affected. Among the six cognitive functions assessed – executive function, visuospatial function, memory, psychomotor speed, attention and language – the influence of CD33 SNPs was most evident in the memory domain among individuals with chronic viral hepatitis. A significant impact on global cognitive functions, executive processes and attention was also noted in individuals with chronic HBV. Notably, the patterns of cognitive variation attributed to CD33 SNPs correlate with the cognitive deficit profiles of Alzheimer’s disease and do not align with Parkinson’s disease-associated cognitive impairments, which predominantly impact executive and visuospatial functions.^24,25^ These results also show the effect of CD33 SNPs to be more specific to the pathogenesis of Alzheimer’s disease, but not to Parkinson’s disease or other neurodegenerative diseases. Although the present study did not include a direct Alzheimer’s disease cohort, the mechanisms we observed in viral hepatitis may parallel CD33’s role in the former condition, particularly in relation to neuroinflammation. This could explain the impact of CD33 SNPs on the cognitive profiles seen in our hepatitis cohorts. These findings suggest that CD33’s influence on neurocognitive decline may extend beyond neurodegenerative diseases such as Alzheimer’s disease to potentially include viral hepatitis.

In this study, we examined five distinct SNPs across three gene blocks of the CD33 gene. All these SNPs exhibited a significant impact on cognitive functions, most notably within the memory domain, in individuals with chronic viral hepatitis. This highlights the phenotypic relevance of each segment of the CD33 gene: rs12985029 is located in the block corresponding to the upstream segment. On the other hand, rs3826656, rs3865444 and rs12459419, which exhibit strong linkage disequilibrium, are situated within the CD33 gene. Conversely, rs33978622 is positioned at the 3′ end relative to the other SNPs.

Although the impact of rs12985029 and rs33978622 on cognitive functions is seldom reported, growing evidence points to an association between Alzheimer’s disease and the three SNPs in linkage disequilibrium. Specifically, the G allele of rs3826656 is associated with Alzheimer’s disease^26^ and correlates with reduced brain volumes and accelerated atrophy rates in Alzheimer’s-related regions among elderly individuals without dementia.^27^ Additionally, rs3865444 has been implicated in the risk of Alzheimer’s disease.^5^ Existing research suggests that the protective nature of its minor allele is attributed to the augmented expression of the short isoform (CD33m), which facilitates Aβ42 phagocytosis.^28,29^ In contrast, the full-length CD33 (CD33M) impedes the uptake and clearance of Aβ42.^2^ The CD33m isoform lacks the sialic acid-binding domain, encoded by exon 2, and its splicing is influenced by rs12459419.^30,31^ Each copy of the minor allele of rs12459419 reduces CD33M expression by approximately 25% and decreases the Alzheimer’s odds ratio by about 0.10.^32^ Notably, research confirms that these SNPs significantly alter CD33 protein levels in human plasma,^33^ reinforcing the profound influence of CD33 SNPs across the CD33 gene, as detailed in our study.

In this study, we observed an augmented influence of CD33 SNPs in the presence of chronic HBV and HCV infections, an effect not observed in Parkinson’s disease, the second most prevalent neurodegenerative disorder. Interestingly, cognitive differences conferred by CD33 SNPs were primarily related to memory, underscoring CD33’s specificity impact on Alzheimer’s disease. Because the pathogenic nature of CD33 in Alzheimer’s disease is often thought to be related to the inhibition of phagocytosis resulting in diminished Aβ clearance and consequent neurodegeneration, the mechanism underlying the enhancement of CD33 SNP effects by HBV and HCV could be linked to phagocytosis inhibition.

Specialised populations of border-associated macrophages and dendritic cells within the CNS coexist with microglia. These populations undergo immunological modifications with ageing and in specific pathological conditions.^34^ Additionally, it has been demonstrated that border-associated macrophages can mediate peripheral immune cell recruitment in an α-synuclein Parkinson’s disease model.^35^ Prior research indicates that the hepatitis B e antigen triggers immune tolerance and hampers T-cell functionality by expanding CD33^+^ monocytic MDSCs, immature myeloid cells that inhibit the immune responses.^36,37^ Similarly, HCV promotes the expansion of CD33^+^ MDSCs via viral RNA-carrying exosomes, leading to immune dysregulation.^10,11,38^ These results suggest potential immunosuppressive or dysregulatory effects of both HBV and HCV, which could further disrupt Aβ clearance in the CNS. Our findings reveal that CD33 SNPs significantly affect CTT1 performance, especially in individuals exhibiting less severe liver inflammation as indicated by the FIB-4 marker. This finding implies that CD33 SNPs’ influence on psychomotor speed – a cognitive domain significantly impacted during Aβ deposition^39^ – is heightened in those with an inhibited inflammatory state. Our results show the interplay between CD33 SNPs and inflammation severity in cognitive dysfunction progression, supporting the hypothesis that chronic viral hepatitis induces an immunosuppressed state, which may further hinder microglial phagocytosis and Aβ clearance, thereby accelerating neurodegeneration. This compounded effect from chronic HBV or HCV infection could exacerbate neurocognitive decline, as indicated by the stronger impact of CD33 SNPs in these individuals. While no direct evidence links HBV or HCV to brain pathology via CNS-initiated events, our findings suggest that systemic immunosuppression associated with these infections might indirectly influence neurodegeneration by modulating CD33^+^ cells – including microglia, peripheral myeloid cells and other CNS-resident macrophages. Thus, both central and peripheral immune mechanisms involving CD33^+^ cells may contribute to the observed neurocognitive decline.

This study acknowledges certain limitations. First, potential selection bias due to demographic variations across different disease groups is noted, yet these variations are consistent with the epidemiological patterns in Taiwan.^40^ Additionally, the nature of Mendelian randomisation in genetic studies should mitigate these discrepancies. Confounding factors such as age, gender and education have been adjusted in our analysis to strengthen the results. Another limitation is the modest number of participants with chronic HBV and HCV, attributed to the extensive time commitment required for neuropsychological testing, which limits the feasibility of investigating the dose–response effect of the CD33 SNPs on cognitive functions. Furthermore, the absence of an independent cohort for external validation, while not critically undermining the study, does limit the ability to fully generalise the findings to other populations. This suggests a direction for future research to enhance the external validity of the study. The absence of the rs201074739 polymorphism in the Eastern Asian population, as confirmed by the ALFA Allele Frequency database, is another limitation of this study. This 4-base pair indel, which disrupts functional CD33, occurs at a 3% minor allele frequency in North American Caucasians but was not present in our study population, so we did not include it in our analysis. Future research should explore other frameshift mutations to better understand the impact of CD33 polymorphisms on cognitive functions.

We intentionally included older adults (>50 years) to better capture cognitive differences influenced by CD33 SNPs and disease status, as this age group is more prone to neurodegenerative pathologies. We also excluded participants with psychiatric disorders, to minimise factors that could confound the relationship between viral hepatitis, CD33 SNPs and cognition. However, these choices limited participant diversity, potentially affecting the generalisability of our findings. Additionally, our assessment of executive functioning and visuospatial functions relied on screening tools with limited sensitivity, which may have missed subtle deficits. Future studies should employ more comprehensive and sensitive neuropsychological batteries, particularly for these domains, to identify subtle or subclinical impairments that may have been overlooked.

The outcomes of this research offer pivotal insights with multifaceted implications. First, they enhance our understanding of the influence exerted by CD33 SNPs on cognitive functions, elucidating CD33’s intricate role in neurodegenerative pathogenesis. Second, the observed interactions between CD33 and chronic viral hepatitis underscoring the profound moderating effect of chronic inflammation on CD33 functions. Our finding not only identifies populations vulnerable to the adverse consequences of enhanced CD33 activity, but also delineates the interplay of genetic and environmental factors in neurocognitive disruptions. Moreover, these insights present promising avenues for therapeutic strategies, advocating the targeted modulation of CD33 in addressing neurocognitive disorders associated with chronic viral hepatitis or sustained inflammation.

In summary, by integrating neuropsychological assessments spanning multiple cognitive domains with genotypic analyses of CD33 SNPs, we identified a pronounced effect of these in individuals afflicted with chronic HBV and HCV – an effect notably absent in Parkinson’s disease individuals. These cognitive function alterations associated with CD33 SNPs were primarily observed in the cognitive domain of memory. Additionally, our data suggest an underlying mechanism, potentially rooted in the inhibited inflammatory state induced by chronic HBV and HCV. Taken together, these findings clarify the interactions between CD33 and chronic HBV and HCV in determining cognitive trajectories, and provide promising therapeutic avenues to combat neurocognitive dysfunctions arising from chronic viral hepatitis or broader neurodegenerative challenges.

## Supporting information

Tsai et al. supplementary material Tsai et al. supplementary material

## Data Availability

The associated data not provided within the paper are available on request from C.-H.T.

## Supplementary material

The supplementary material can be found online at https://doi.org/10.1192/bjo.2025.10048
