# Supplementary material for: Association of CD33 genetic variants with neurocognitive profiles in chronic viral hepatitis
Source: BJPsych Open. 2025 Jul 10;11(4):e147. doi: 10.1192/bjo.2025.10048 (PMC12247067; doi:10.1192/bjo.2025.10048)
Supplement: Tsai et al. supplementary material [file S2056472425100483sup001.pdf]

Supplementary Table 1. The allelic frequency of CD33 SNPs in all participants.

SNP ID	Position	Minor/ Major allele	Most severe consequence	MAF (%)	HWE (p-value)	Population Alt allele frequency			
						Alt allele	Ref allele	ALFA EAS	ALFA total
rs12985029	51210109	A/G	2KB Upstream Variant	21.347%	χ^2=0.415 (p=0.520)	A	G	0.23	0.3006
rs3987765	51210487	T/G	2KB Upstream Variant	0.104%	χ^2=0.001 (p=0.974)	T	G	0.00	0.0597
rs116999688	51211363	C/G	5 Prime UTR Variant	0.835%	χ^2=0.068 (p=0.794)	C	G	0.00	0.0002
rs3826656	51223357	A/G	Intron variant	26.510%	χ^2= 0.167 (p=0.683)	A	G	0.2950	0.7452
rs1710398	51223655	C/A	Intron variant	7.411%	χ^2=0.015 (p=0.902)	C	A	0.062	0.4239
rs3865444	51224706	A/C	5 Prime UTR Variant	17.207%	χ^2=2.751 (p=0.097)	A	C	0.1923	0.3023
rs12459419	51225221	T/C	missense variant	17.240%	χ^2=2.183 (p=0.140)	T	C	0.109	0.2638
rs2455069	51225385	G/A	missense variant	7.411%	χ^2=0.015 (p=0.902)	G	A	0.015	0.3957
rs80165437	51232408	A/G	Intron variant	3.086%	χ^2=1.389 (p=0.239)	A	G	0.03	0.0004
rs33978622	51233128	C/G	Intron variant	20.241%	χ^2= 0.055 (p=0.815)	C	G	0.20	0.2960
rs111722079	51235676	A/G	Synonymous Variant	0.000%	-	A	G	0.0000	0.0009

Abbreviations: SNP = single nucleotide polymorphism, MAF = minor allele frequency,

HWE = Hardy-Weinberg Equilibrium, ALFA = Allele frequency aggregator, EAS = East Asians.

Supplementary Table 2. Demographic Characteristics and Neuropsychological Test Scores by Study Group.

				Control	HBV	HCV	PD	Kruskal-Wallis p-value	Between Group
				Mean ± SD	Mean ± SD	Mean ± SD	Mean ± SD		
Number				238	61	93	171		
Age (years)				64.59 ± 6.634	60.18 ± 6.297	62.75 ± 7.681	65.70 ± 7.430	p<0.001*	NC-PD 0.476 NC-HBV <0.001*<br>NC-HCV 0.208 PD-HBV <0.001*<br>PD-HCV 0.015 HBV-HCV 0.178
Male/Female				64/174	44/17	33/60	115/56	p<0.001*	NC-PD <0.001* NC-HBV <0.001*<br>NC-HCV 0.506 PD-HBV 0.917<br>PD-HCV <0.001* HBV-HCV <0.001*
Education (years)				12.7 9± 3.451	13.28 ± 3.215	10.68 ± 3.636	12.44 ± 3.624	p<0.001*	NC-PD 0.795 NC-HBV 0.817<br>NC-HCV <0.001* PD-HBV 0.460<br>PD-HCV 0.002* HBV-HCV <0.001*
AST (IU/L)				-	31.33 ± 21.312	36.02 ± 30.140	-	p=0.198‡	
ALT (IU/L)				-	30.46 ± 23.408	34.17 ± 29.222	-	p=0.647‡	
HBV antiviral use / no				-	36/25	-	-		
JCV Treatment-naive / SVR				-	-	26/67	-		
FIB-4 index				-	1.93 ± 0.894	2.39 ± 1.545	-	p=0.072‡	
Global cognitive screening	Mini-Mental State Examination			27.50 ± 2.116	28.15 ± 1.289	27.27 ± 2.127	26.73 ± 2.270	p=0.003<br>**q=0.004***	NC-PD 0.122 NC-HBV 0.222<br>NC-HCV 0.939 PD-HBV 0.004*<br>PD-HCV 0.655 HBV-HCV 0.153
Executive function	Color Trails Test 2			109.53 ± 43.749	100.44 ± 40.778	124.47 ± 57.553	132.07 ± 61.622	p<0.001<br>**q<0.001***	NC-PD <0.001* NC-HBV 0.733<br>NC-HCV 0.202 PD-HBV <0.001*<br>PD-HCV 0.243 HBV-HCV 0.084
Visuospatial function	Pentagons Copy (MMSE)			0.91 ± 0.283	0.89 ± 0.321	0.83 ± 0.379	0.88 ± 0.324	p=0.286<br>q=0.286	
Memory	Wechsler Memory Scale-III	Logical Memory	Immediate	34.86 ± 10.875	33.08 ± 10.057	27.82 ± 10.847	29.95 ± 12.221	p<0.001<br>**q<0.001***	NC-PD <0.001* NC-HBV 0.842<br>NC-HCV <0.001* PD-HBV 0.174<br>PD-HCV 0.814 HBV-HCV 0.053
			Delayed	21.69 ± 8.598	19.92 ± 8.233	15.96 ± 7.492	16.95 ± 9.730	p<0.001<br>**q<0.001**	NC-PD <0.001* NC-HBV 0.711<br>NC-HCV <0.001* PD-HBV 0.080<br>PD-HCV 0.980 HBV-HCV 0.065
		Visual Reproduction	Immediate	74.37 ± 14.832	75.46 ± 12.461	67.44 ± 15.548	69.13 ± 16.234	p<0.001*<br>**q<0.001***	NC-PD <0.001* NC-HBV 0.974<br>NC-HCV 0.006* PD-HBV 0.006*<br>PD-HCV 0.997 HBV-HCV 0.027*
			Delayed	51.72 ± 20.928	54.54 ± 19.551	43.22 ± 22.638	43.01 ± 24.943	p<0.001<br>**q<0.001***	NC-PD 0.001* NC-HBV 0.853<br>NC-HCV 0.022* PD-HBV 0.004*<br>PD-HCV 0.993 HBV-HCV 0.023*
Psychomotor speed	Color Trails Test 1			52.15 ± 22.089	46.93 ± 22.849	53.90 ± 27.738	64.95 ± 33.557	p<0.001<br>**q<0.001***	NC-PD <0.001* NC-HBV 0.724<br>NC-HCV 0.976 PD-HBV <0.001*<br>PD-HCV0.001* HBV-HCV0.615
Attention	Paced Auditory Serial Additional Test			73.17 ± 17.313	75.90 ± 12.587	65.04 ± 20.524	65.56 ± 18.265	p<0.001<br>**q<0.001***	NC-PD 0.013* NC-HBV 0.675<br>NC-HCV 0.006* PD-HBV 0.006*<br>PD-HCV 1.000 HBV-HCV 0.004*
Language	Language (MMSE)			4.85 ± 0.406	4.72 ± 0.452	4.88 ± 0.357	4.91 ± 0.294	p=0.082<br>q=0.091	

The q-value used here is the False Discovery Rate (FDR), employed to correct the results of multiple comparisons involving 5 SNPs. * *q<0.05*. ‡ Mann-Whitney U test.

Supplementary Table 3A. Effects of CD33 SNPs on the cognitive functions of participants in the healthy control group

SNP				rs12985029 (n=235)				rs3826656 (n=237)				rs33978622 (n=237)			
Genotype				AA (n=13)	AG (n=74)	GG (n=148)	Statistic	AA (n=20)	AG (n=112)	GG (n=125)	Statistic	CC (n=11)	CG (n=67)	GG (n=159)	Statistic
Age (years)				61.15 ±8.305	64.41 ±7.241	64.82 ±6.015	p=0.156	60.85 ±7.666	64.73 ±6.797	65.07 ±6.221	p=0.029*<br>AA-AG 0.059<br>AA-GG 0.030<br>AG-GG 0.930	61.27 ±7.198	64.00 ±7.473	65.06 ±6.186	p=0.132
Male/Female				4/9	14/60	45/103	p=0.326	5/15	22/90	37/88	p=0.636	2/9	16/51	46/113	p=0.590
Education (years)				12.85 ±1.625	12.59 ±3.864	12.88 ±3.391	p=0.950	13.40 ±2.722	12.88 ±3.784	12.62 ±3.314	p=0.610	13.36 ±1.804	12.79 ±3.422	12.74 ±3.564	p=0.846
Global cognitive screening	Mini-Mental State Examination			27.77 ±1.363	27.20 ±2.094	27.53 ±2.254	p=0.644 q=0.968	27.15 ±2.007	27.38 ±1.815	27.54 ±2.408	p=0.700 q=0.968	26.91 ±2.023	27.45 ±1.520	27.48 ±2.389	p=0.696 q=0.968
Executive function	Color Trails Test 2			101.66 ±41.906	109.25 ±44.918	110.13 ±42.926	p=0.798 q=0.968	103.45 ±50.150	109.18 ±44.886	110.76 ±41.322	P=0.781 q=0.968	95.47 ±40.025	104.25 ±41.691	112.73 ±44.108	p=0.222 q=0.968
Visuospatial function	Pentagons Copy (MMSE)			0.85 ±0.376	0.88 ±0.329	0.91 ±0.284	p=0.717 q=0.968	0.80 ±0.410	0.90 ±0.299	0.91 ±0.284	p=0.305 q=0.968	1.00 ±0.000	0.90 ±0.308	0.89 ±0.310	p=0.525 q=0.968
Memory	Wechsler Memory Scale-III	Logical Memory	Immediate	32.15 ±9.634	34.23 ±11.455	35.05 ±10.900	p=0.713 q=0.968	34.15 ±12.171	34.47 ±10.413	34.74 ±11.240	p=0.968 q=0.968	36.55 ±9.070	34.22 ±9.395	34.60 ±11.707	p=0.810 q=0.968
			Delayed	20.46 ±9.632	21.07 ±9.383	21.74 ±8.312	p=0.835 q=0.968	21.35 ±11.431	21.24 ±8.364	21.57 ±8.484	p=0.962 q=0.968	24.73 ±7.669	21.06 ±8.565	21.35 ±8.794	p=0.424 q=0.968
		Visual Reproduction	Immediate	74.33 ±9.847[a]	75.56 ±16.554[b]	73.75 ±14.324[b]	p=0.858 q=0.968	72.89 ±14.670	75.93 ±15.497[b]	73.33 ±14.354[b]	p=0.418 q=0.968	78.18 ±11.488	74.43 ±14.232[b]	73.97 ±15.300[c]	p=0.660 q=0.968
			Delayed	51.58 ±20.322[a]	51.13 ±19.377[b]	51.88 ±21.894[b]	p=0.948 q=0.968	52.42 ±17.131[a]	51.09 ±20.475[b]	52.09 ±21.991[b]	p=0.934 q=0.968	55.45 ±16.476	52.03 ±20.508[b]	51.34 ±21.523[c]	p=0.815 q=0.968
Psychomotor speed	Color Trails Test 1			43.47 ±16.257	51.46 ±18.177	53.22 ±23.987	p=0.480 q=0.968	43.90 ±14.852	52.24 ±19.261	53.42 ±24.365	p=0.196 q=0.968	41.02 ±11.158	49.92 ±18.085	53.87 ±23.617	p=0.104 q=0.968
Attention	Paced Auditory Serial Addition Test			67.00 ±23.295	72.51 ±17.256	73.23 ±17.416	p=0.609 q=0.968	72.75 ±20.614	74.37 ±17.074	71.50 ±17.671	p=0.498 q=0.968	68.18 ±24.008	73.18 ±16.445	72.84 ±17.772	p=0.680 q=0.968
Language	Language (MMSE)			4.92 ±0.277	4.82 ±0.449	4.85 ±0.393	p=0.769 q=0.968	5.00 ±0.000	4.82 ±0.443	4.85 ±0.403	p=0.180 q=0.968	5.00 ±0.000	4.85 ±0.399	4.84 ±0.419	p=0.432 q=0.968

The q-value used here is the False Discovery Rate (FDR), employed to correct the results of multiple comparisons involving 5 SNPs.

[a]One missing value. [b]Four missing values. [c]Five missing values.

Supplementary Table 3B: Effects of rs3865444 and rs12459419 on the cognitive functions of participants in the healthy control group

SNP				rs3865444(n=235)			rs12459419 (n=237)		
Genotype				AA+AC (n=76)	CC (n=159)	Statistic	TT+TC (n=77)	CC (n=160)	Statistic
Age (years)				64.38±7.517	64.61±6.163	p=0.728	64.19±7.645	64.77±6.128	p=0.531
Male/Female				16/60	47/112	p=0.169	16/61	48/112	p=0.135
Education (years)				12.87±3.388	12.75±3.517	p=0.806	12.88±3.368	12.73±3.506	p=0.740
Global cognitive screening	Mini-Mental State Examination			27.37±1.839	27.49±2.300	p=0.276 q=0.917	27.36±1.827	27.49±2.304	p=0.256 q=0.917
Executive function	Color Trails Test 2			104.67±42.919	111.21±43.443	p=0.121 q=0.917	104.30±42.763	112.05±43.559	p=0.070 q=0.917
Visuospatial function	Pentagons Copy (MMSE)			0.89±0.309	0.90±0.302	p=0.913 q=0.988	0.90±0.307	0.90±0.301	p=0.926 q=0.988
Memory	Wechsler Memory Scale-III	Logical Memory	Immediate	34.05±9.384	34.97±11.538	p=0.366 q=0.917	34.19±9.405	34.77±11.660	p=0.488 q=0.917
			Delayed	21.09±8.083	21.73±8.856	p=0.450 q=0.917	21.22±8.109	21.52±8.965	p=0.638 q=0.938
		Visual Reproduction	Immediate	75.00±13.792[a]	74.11±15.167[b]	p=0.635 q=0.938	75.17±13.768[a]	73.90±15.312[b]	p=0.519 q=0.917
			Delayed	51.32±18.245[a]	52.15±21.985[b]	p=0.866 q=0.988	51.53±18.198[a]	51.83±22.191[b]	p=0.997 q=0.997
Psychomotor speed	Color Trails Test 1			49.61±18.427	53.38±23.445	p=0.269 q=0.917	49.53±18.322	53.43±23.368	p=0.233 q=0.917
Attention	Paced Auditory Serial Addition Test			72.08±18.155	73.06±17.438	p=0.710 q=0.975	72.34±18.177	72.90±17.489	p=0.850 q=0.988
Language	Language (MMSE)			4.87±0.377	4.84±0.419	p=0.580 q=0.917	4.87±0.375	4.84±0.418	p=0.567 q=0.917

The q-value used here is the False Discovery Rate (FDR), employed to correct the results of multiple comparisons involving 5 SNPs.

[a]Five missing value. [b]Four missing value.

Supplementary Table 4A. Effects of CD33 SNPs on the cognitive functions of participants with chronic viral hepatitis B

SNP				rs12985029 (n=61)				rs3826656 (n=61)				rs33978622 (n=61)			
Genotype				AA<br>(n=2)	AG<br>(n=19)	GG<br>(n=40)	Statistic	AA<br>(n=2)	AG<br>(n=24)	GG<br>(n=35)	Statistic	CC<br>(n=6)	CG<br>(n=13)	GG<br>(n=42)	Statistic
Age (years)				57.50<br>±2.121	58.58<br>±4.970	61.08<br>±6.859	p=0.306	56.50<br>±3.536	59.54<br>±5.703	60.83<br>±6.789	p=0.530	57.00<br>±3.521	59.15<br>±5.336	60.95<br>±6.764	p=0.290
Male/Female				2/0	13/6	29/11	p=0.648	2/0	17/7	25/10	p=0.681	4/2	11/2	29/13	p=0.537
Education (years)				13.00<br>±1.414	14.42<br>±2.652	12.75<br>±3.418	p=0.175	13.00<br>±1.414	13.75<br>±9.326	12.97<br>±3.408	p=0.661	15.50<br>±2.168	14.77<br>±2.891	12.50<br>±3.172	p=0.015*<br>CC-CG 0.888<br>CC-GG 0.086<br>CG-GG 0.071
Global cognitive screening	Mini-Mental State Examination			29.50<br>±0.707	28.63<br>±1.116	27.85<br>±1.292	p=0.027<br>q=0.090<br>AA-AG 0.640<br>AA-GG 0.190<br>AG-GG 0.083	28.00<br>±1.414	28.46<br>±1.285	27.94<br>±1.282	p=0.321<br>q=0.357	28.67<br>±1.033	28.77<br>±1.235	27.88<br>±1.273	p=0.053<br>q=0.122
Executive function	Color Trails Test 2			64.32<br>±2.680	89.94<br>±31.701	107.23<br>±43.890	p=0.140<br>q=0.221	87.59<br>±35.596	85.36<br>±28.868	111.52<br>±45.133	p=0.046<br>q=0.115<br>AA-AG 0.997<br>AA-GG 0.706<br>AG-GG 0.050	72.25<br>±23.782	89.37<br>±31.518	107.89<br>±43.148	p=0.071<br>q=0.142
Visuospatial function	Pentagons Copy (MMSE)			1.00<br>±0.000	0.95<br>±0.229	0.85<br>±0.362	p=0.493<br>q=0.510	1.00<br>±0.000	0.96<br>±0.204	0.83<br>±0.382	p=0.279<br>q=0.330	1.00<br>±0.000	1.00<br>±0.000	0.83<br>±0.377	p=0.173<br>q=0.260
Memory	Wechsler Memory Scale-III	Logical Memory	Immediate	29.50<br>±0.707	40.53<br>±9.571	29.73<br>±8.608	p<0.001*<br>**q=0.004***<br>AA-AG 0.253<br>AA-GG 0.999<br>AG-GG <0.001	33.00<br>±5.657	38.08<br>±10.866	29.66<br>±8.242	p=0.005<br>**q=0.021***<br>AA-AG 0.762<br>AA-GG 0.886<br>AG-GG 0.005	44.50<br>±8.803	37.08<br>±9.604	30.21<br>±8.888	p=0.001<br>**q=0.008***<br>CC-CG 0.258<br>CC-GG 0.003<br>CG-GG 0.065
			Delayed	17.00<br>±1.414	25.95<br>±8.010	17.20<br>±6.992	p<0.001<br>**q=0.004***<br>AA-AG 0.262<br>AA-GG 0.999<br>AG-GG <0.001	20.50<br>±6.364	23.13<br>±9.289	17.69<br>±6.902	p=0.042<br>q=0.115<br>AA-AG 0.904<br>AA-GG 0.888<br>AG-GG 0.042	28.00<br>±7.239	23.31<br>±8.380	17.71<br>±7.353	p=0.003<br>**q=0.015***<br>CC-CG 0.459<br>CC-GG 0.011<br>CG-GG0.075
		Visual Reproduction	Immediate	83.50<br>±9.192	79.42<br>±11.087	73.18<br>±12.794	p=0.129<br>q=0.221	81.50<br>±12.021	78.92<br>±10.508	72.74<br>±13.300	p=0.137<br>q=0.221	82.50<br>±14.181	79.08<br>±9.491	73.33<br>±12.662	p=0.120<br>q=0.221
			Delayed	65.00<br>±0.000	67.21<br>±18.573	48.00<br>±17.355	P=0.001<br>**q=0.008***<br>AA-AG 0.986<br>AA-GG 0.417<br>AG-GG 0.001	55.50<br>±13.435	62.33<br>±20.160	49.14<br>±17.928	p=0.036<br>q=0.108<br>AA-AG 0.885<br>AA-GG 0.897<br>AG-GG0.036	72.33<br>±20.393	63.46<br>±16.801	49.24<br>±18.047	p=0.003<br>**q=0.015***<br>CC-CG 0.610<br>CC-GG 0.018<br>CG-GG 0.053
Psychomotor speed	Color Trails Test 1			30.09<br>±1.068	42.38<br>±11.689	49.94<br>±26.574	p=0.286<br>q=0.330	36.89<br>±8.556	41.38<br>±11.130	51.32<br>±28.069	p=0.216<br>q=0.295	36.83<br>±13.180	41.60<br>±11.296	50.03<br>±25.917	p=0.270<br>q=0.330
Attention	Paced Auditory Serial Addition Test			91.50<br>±0.707	80.37<br>±10.062	73.00<br>±12.890	p=0.020<br>**q=0.075***<br>AA-AG 0.462<br>AA-GG 0.112<br>AG-GG 0.096	85.00<br>±9.899	78.21±10.644	73.80<br>±13.670	p=0.247<br>q=0.322	86.50<br>±10.015	77.69<br>±12.358	73.83<br>±12.372	p=0.057<br>q=0.122
Language	Language (MMSE)			5.00<br>±0.000	4.84<br>±0.375	4.65<br>±0.483	p=0.213<br>q=0.295	5.00<br>±0.000	4.71<br>±0.464	4.71<br>±0.458	p=0.681<br>q=0.681	4.83<br>±0.408	4.85<br>±0.376	4.67<br>±0.477	p=0.379<br>q=0.406

The q-value used here is the False Discovery Rate (FDR), employed to correct the results of multiple comparisons involving 5 SNPs. * *q<0.05.*

Supplementary Table 4B: Effects of rs3865444 and rs12459419 on the cognitive functions of individuals with chronic viral hepatitis B infection

SNP				rs3865444(n=61)			rs12459419 (n=61)		
Genotype				AA+AC (n=18)	CC (n=43)	Statistic	TT+TC (n=18)	CC (n=43)	Statistic
Age (years)				58.33±4.899	60.95±6.697	p=0.175	58.33±4.899	60.95±6.697	p=0.175
Male/Female				14/4	30/13	p=0.528	14/4	30/13	p=0.528
Education (years)				14.44±2.640	12.79±3.335	p=0.077	14.44±2.640	12.79±3.335	p=0.077
Global cognitive screening	Mini-Mental State Examination			28.78±1.166	27.88±1.258	p=0.013 **q=0.039***	28.78±1.166	27.88±1.258	p=0.013 **q=0.039***
Executive function	Color Trails Test 2			83.47±30.630	107.55±42.669	p=0.015 **q=0.039***	83.47±30.630	107.55±42.669	p=0.015 **q=0.039***
Visuospatial function	Pentagons Copy (MMSE)			0.94±0.236	0.86±.0.351	p=0.352 q=0.359	0.94±0.236	0.86±0.351	p=0.352 q=0.359
Memory	Wechsler Memory Scale-III	Logical Memory	Immediate	40.11±9.222	30.14±8.946	p=0.001 **q=0.010***	40.11±9.222	30.14±8.946	p=0.001 **q=0.010***
			Delayed	24.89±8.203	17.84±7.387	p=0.003 **q=0.012***	24.89±8.203	17.84±7.387	p=0.003 **q=0.012***
		Visual Reproduction	Immediate	80.06±11.196	73.53±12.580	p=0.064 q=0.089	80.06±11.196	73.53±12.580	p=0.064 q=0.089
			Delayed	66.89±17.769	49.37±18.044	p=0.002 **q=0.010***	66.89±17.769	49.37±18.044	p=0.002 **q=0.010***
Psychomotor speed	Color Trails Test 1			39.08±10.789	50.22±25.714	p=0.050 q=0.080	39.08±10.789	50.22±25.714	p=0.050 q=0.080
Attention	Paced Auditory Serial Addition Test			81.11±10.927	73.72±12.708	p=0.037 q=0.071	81.11±10.927	73.72±12.708	p=0.037 q=0.071
Language	Language (MMSE)			4.83±0.383	4.67±0.474	p=0.211 q=0.239	4.83±0.383	4.67±0.474	p=0.211 q=0.239

The q-value used here is the False Discovery Rate (FDR), employed to correct the results of multiple comparisons involving 5 SNPs. * *q<0.05*.

Supplementary Table 5A. Effects of CD33 SNPs on the cognitive functions of participants with chronic viral hepatitis C

SNP				rs12985029 (n=93)				rs3826656 (n=93)				rs33978622 (n=93)			
Genotype				AA<br>(n=2)	AG<br>(n=41)	GG<br>(n=50)	Statistic	AA<br>(n=6)	AG<br>(n=42)	GG<br>(n=45)	Statistic	CC<br>(n=5)	CG<br>(n=36)	GG<br>(n=52)	Statistic
Age (years)				66.50<br>±14.849	62.37<br>±7.654	62.92<br>±7.597	p=0.744	66.33<br>±10.053	62.69<br>±7.469	62.33<br>±7.613	p=0.491	69.00<br>±7.874	62.44<br>±7.109	62.37<br>±7.926	p=0.175
Male/Female				1/1	14/27	18/32	p=0.898	3/3	11/31	19/26	p=0.225	2/3	13/23	18/34	p=0.968
Education (years)				6.50<br>±0.707	10.05<br>±3.383	11.36<br>±3.735	p=0.058	9.67<br>±2.733	10.26<br>±3.321	11.20<br>±3.992	p=0.383	8.20<br>±4.382	10.83<br>±3.291	10.81<br>±3.778	p=0.296
Global cognitive screening	Mini-Mental State Examination			26.00<br>±1.414	27.37<br>±1.813	27.24<br>±2.387	p=0.673<br>q=0.841	27.33<br>±1.862	27.33<br>±2.068	27.20<br>±2.252	p=0.956<br>q=0.989	27.20<br>±1.483	27.28<br>±1.876	27.27<br>±2.361	p=0.997<br>q=0.997
Executive function	Color Trails Test 2			98.78<br>±6.251	120.72<br>±41.903	128.58<br>±68.769	p=0.666<br>q=0.841	103.32<br>±39.469	123.51<br>±51.711	128.19<br>±64.661	p=0.608<br>q=0.841	114.31<br>±41.688	111.56<br>±40.146	134.39<br>±67.117	p=0.173<br>q=0.656
Visuospatial function	Pentagons Copy (MMSE)			0.50<br>±0.707	0.83<br>±0.381	0.84<br>±0.370	p=0.467<br>q=0.808	0.83<br>±0.408	0.81<br>±0.397	0.84<br>±0.367	p=0.913<br>q=0.989	1.00<br>±0.000	0.81<br>±0.401	0.83<br>±0.382	p=0.567<br>q=0.841
Memory	Wechsler Memory Scale-III	Logical Memory	Immediate	24.50<br>±7.778	30.17<br>±10.758	26.02<br>±10.807	p=0.175<br>q=0.656	30.00<br>±15.362	30.33<br>±10.515	25.18<br>±10.116	p=0.074<br>q=0.444	18.60<br>±11.887	30.06<br>±9.692	27.15<br>±11.152	p=0.068<br>q=0.444
			Delayed	11.50<br>±0.707	17.76<br>±7.388	14.66<br>±7.455	p=0.101<br>q=0.505	17.33<br>±10.367	18.50<br>±7.075	13.40<br>±6.737	p=0.005<br>q=0.105<br>AA-AG 0.997<br>AA-GG 0.583<br>AG-GG 0.084	9.60<br>±5.367	18.08<br>±7.064	15.10<br>±7.539	p=0.026<br>q=0.260<br>CC-CG 0.055<br>CC-GG 0.277<br>CG-GG 0.172
		Visual Reproduction	Immediate	73.50<br>±2.121	69.85<br>±13.363	65.22<br>±17.223	p=0.318<br>q=0.795	73.33<br>±10.386	68.38<br>±15.041	65.78<br>±16.553	p=0.470<br>q=0.808	62.80<br>±11.649	70.97<br>±12.557	65.44<br>±17.385	p=0.207<br>q=0.690
			Delayed	60.50<br>±13.435	44.34<br>±20.303	41.60<br>±24.619	p=0.472<br>q=0.808	43.67<br>±19.735	44.24<br>±21.151	42.20<br>±24.665	p=0.916<br>0.989	31.80<br>±28.446	45.58<br>±20.276	42.67<br>±23.703	p=0.433<br>q=0.808
Psychomotor speed	Color Trails Test 1			46.00<br>±8.146	55.95<br>±29.431	52.54<br>±26.985	p=0.780<br>q=0.936	53.09<br>±18.900	55.16<br>±29.703	52.84<br>±27.263	p=0.926<br>q=0.989	54.72<br>±14.542	49.12<br>±17.292	57.13<br>±33.754	p=0.415<br>q=0.808
Attention	Paced Auditory Serial Addition Test			51.50<br>±6.364	65.95<br>±18.720	64.84<br>±22.265	p=0.625<br>q=0.841	74.33<br>±20.304	63.93<br>±17.979	64.84<br>±22.796	p=0.512<br>q=0.808	61.40<br>±19.957	68.14<br>±19.624	63.25<br>±21.283	p=0.508<br>q=0.808
Language	Language (MMSE)			4.50<br>±0.707	4.88<br>±0.400	4.90<br>±0.303	p=0.300<br>q=0.795	4.67<br>±0.516	4.90<br>±0.370	4.89<br>±0.318	p=0.308<br>q=0.795	4.40<br>±0.548	4.92<br>±0.368	4.90<br>±0.298	p=0.007<br>q=0.105<br>CC-CG 0.008<br>CC-GG 0.009<br>CG-GG 0.985

The q-value used here is the False Discovery Rate (FDR), employed to correct the results of multiple comparisons involving 5 SNPs.

Supplementary Table 5B: Effects of rs3865444 and rs12459419 on the cognitive functions of participants with chronic viral hepatitis C

SNP				rs3865444(n=92)			rs12459419 (n=93)		
Genotype				AA+AC<br>(n=36)	CC<br>(n=56)	Statistic	TT+TC<br>(n=36)	CC<br>(n=57)	Statistic
Age (years)				64.00±7.772	62.13±7.556	p=0.254	64.00±7.772	61.96±7.585	p=0.214
Male/Female				12/24	21/35	p=0.686	12/24	21/36	p=0.732
Education (years)				10.00±3.243	11.05±3.844	p=0.245	10.00±3.243	11.11±3.830	p=0.207
Global cognitive screening	Mini-Mental State Examination			27.28±1.907	27.29±2.286	p=0.542<br>q=0.934	27.28±1.907	27.26±2.272	p=0.611<br>q=0.970
Executive function	Color Trails Test 2			118.59±44.175	129.38±64.649	p=0.660<br>q=0.970	118.59±44.175	128.19±64.698	p=0.770<br>q=0.970
Visuospatial function	Pentagons Copy (MMSE)			0.86±0.351	0.82±0.386	p=0.617<br>q=0.970	0.86±0.351	0.81±0.398	p=0.503<br>q=0.934
Memory	Wechsler Memory Scale-III	Logical Memory	Immediate	28.92±10.981	26.73±10.484	p=0.213<br>q=0.874	28.92±10.981	27.12±10.800	p=0.278<br>q=0.874
Memory	Wechsler Memory Scale-III	Logical Memory	Delayed	17.58±7.587	14.70±7.160	p=0.056<br>q=0.560	17.58±7.587	14.93±7.312	p=0.075<br>q=0.625
Memory	Wechsler Memory Scale-III	Visual Reproduction	Immediate	69.42±12.949	65.82±16.897	p=0.357<br>q=0.874	69.42±12.949	66.19±16.978	p=0.425<br>q=0.874
Memory	Wechsler Memory Scale-III	Visual Reproduction	Delayed	42.75±20.954	42.79±23.394	p=0.908<br>q=0.970	42.75±20.954	43.51±23.818	p=0.804<br>q=0.970
Psychomotor speed	Color Trails Test 1			56.71±31.282	52.47±25.482	p=0.370<br>q=0.874	56.71±31.282	52.13±25.381	p=0.328<br>q=0.874
Attention	Paced Auditory Serial Addition Test			65.08±19.315	64.71±21.491	p=0.848<br>q=0.970	65.08±19.315	65.02±21.421	p=0.912<br>q=0.970
Language	Language (MMSE)			4.83±0.447	4.91±0.288	p=0.436<br>q=0.874	4.83±0.447	4.91±0.285	p=0.419<br>q=0.874

The q-value used here is the False Discovery Rate (FDR), employed to correct the results of multiple comparisons involving 5 SNPs.

Supplementary Table 6A. Effects of CD33 SNPs on the cognitive functions of participants with PD

SNP				rs12985029 (n=171)				rs3826656 (n=171)				rs33978622 (n=170)			
Genotype				AA (n=12)	AG (n=53)	GG (n=106)	Statistic	AA (n=10)	AG (n=71)	GG (n=90)	Statistic	CC (n=3)	CG (n=66)	GG (n=101)	Statistic
Age (years)				64.83±8.032	64.57±6.784	66.37±7.659	p=0.309	65.90±7.505	66.39±7.283	65.13±7.572	p=0.581	64.67±12.583	65.53±6.678	65.82±61.588	p=0.943
Male/Female				8/4	34/19	73/33	p=0.907	8/2	46/25	61/29	p=0.669	1/2	41/25	73/28	p=0.174
Education (years)				11.17±3.664	12.37±3.528	12.61±3.671	p=0.418	10.30±3.234	12.13±3.674	12.91±3.549	p=0.061	8.00±3.464	11.96±3.522	12.86±3.617	p=0.030*<br>CC-CG 0.175<br>CC-GG 0.071<br>CG-GG 0.286
Global cognitive screening	Mini-Mental State Examination			25.58±3.579	27.47±2.224	26.75±3.005	p=0.070<br>q=0.769	25.70±2.751	26.90±3.113	27.01±2.650	p=0.389<br>q=0.769	26.00±1.732	26.73±3.363	27.04±2.522	p=0.680<br>q=0.916
Executive function	Color Trails Test 2			117.35±43.298[d]	134.28±72.428[c]	144.66±79.899[e]	p=0.467<br>q=0.787	144.43±64.758	145.41±89.063[i]	134.45±65.281[e]	p=0.682<br>q=0.916	127.30±62.450	137.31±59.842[h]	141.47±85.525[j]	p=0.912<br>q=0.916
Visuospatial function	Pentagons Copy (MMSE)			0.75±0.452	0.91±0.295	0.84±0.369	p=0.331<br>q=0.769	0.90±0.316	0.85±0.364	0.86±0.354	p=0.890<br>q=0.916	1.00±0.000	0.86±0.346	0.84±0.367	p=0.715<br>q=0.916
Memory	Wechsler Memory Scale-III	Logical Memory	Immediate	27.33±12.809	29.00±13.512	29.60±12.797	p=0.825<br>q=0.916	21.70±9.764	28.00±12.756	31.09±13.121	p=0.050<br>q=0.769<br>AA-AG 0.366<br>AA-GG 0.093<br>AG-GG 0.293	28.67±5.508	27.48±13.356	30.36±12.849	p=0.378<br>q=0.769
			Delayed	13.92±9.268	16.77±11.067	16.58±9.810	p=0.675<br>q=0.916	11.80±7.376	15.52±10.315	17.70±10.138	p=0.128<br>q=0.769	18.33±6.110	15.12±10.827	17.23±9.802	p=0.405<br>q=0.769
		Visual Reproduction	Immediate	68.40±16.236[d]	67.88±19.146[d]	66.42±18.894[b]	p=0.916<br>q=0.916	67.10±16.176	64.70±19.862[b]	68.78±18.095[b]	p=0.353<br>q=0.769	56.67±23.861	66.28±18.152[c]	67.55±19.028[m]	p=0.585<br>q=0.916
			Delayed	38.10±19.779[d]	46.65±25.581[d]	40.64±24.042[b]	p=0.358<br>q=0.769	31.10±18.150	40.49±25.312[b]	45.13±23.920[b]	p=0.148<br>q=0.769	20.00±12.490	39.93±24.666[c]	44.30±24.101[m]	p=0.154<br>q=0.769
Psychomotor speed	Color Trails Test 1			76.96±63.055[a]	61.63±28.775[c]	74.70±49.860[h]	p=0.256<br>q=0.769	75.17±42.573	72.00±49.832[c]	69.41±42.953[j]	p=0.893<br>q=0.916	82.38±69.583	77.78±59.978[c]	66.07±32.236[j]	p=0.276<br>q=0.769
Attention	Paced Auditory Serial Addition Test			60.57±13.352[c]	65.83±19.078[f]	65.71±18.672[g]	p=0.753<br>q=0.916	54.17±17.759[b]	64.65±18.050[k]	67.47±18.371[l]	p=0.248<br>q=0.769	43.00±0.000[d]	65.88±18.013[n]	65.68±18.633[g]	p=0.472<br>q=0.787
Language	Language (MMSE)			4.67±0.492	4.94±0.233	4.84±0.461	p=0.082<br>q=0.769	4.70±0.483	4.89±0.361	4.86±0.439	p=0.410<br>q=0.769	5.00±0.000	4.85±0.504	4.86±0.347	p=0.821<br>q=0.916

The q-value used here is the False Discovery Rate (FDR), employed to correct the results of multiple comparisons involving 5 SNPs.
[b]Four missing values.[c]Five missing values. [d]Two missing values. [e]Ten missing values. [f] Twenty nine missing values. [g]Fifty one missing values. [h]Eight missing values. [i]Seven one missing values. [j]Nine missing values. [k]Thirty four missing values. [l]Fourty seven missing values. [m]Three missing values. [n]Thirty two missing values.

Supplementary Table 6B: Effects of rs3865444 and rs12459419 on the cognitive functions of participants with PD.

SNP				rs3865444(n=171)			rs12459419 (n=171)		
Genotype				AA+AC (n=59)	CC (n=112)	Statistic	TT+TC (n=59)	CC (n=112)	Statistic
Age (years)				65.20±6.614	65.96±7.842	p=0.791	65.20±6.614	65.96±7.842	p=0.791
Male/Female				36/23	79/33	p=0.209	36/23	79/33	p=0.209
Education (years)				11.87±3.587	12.73±3.624	p=0.104	11.87±3.587	12.73±3.624	p=0.104
Global cognitive screening	Mini-Mental State Examination			27.03±2.710	26.81±2.939	p=0.685 q=0.918	27.03±2.710	26.81±2.939	p=0.685 q=0.918
Executive function	Color Trails Test 2			136.89±70.18[b]	141.19±79.025[c]	p=0.947 q=0.980	136.89±70.185[b]	141.19±79.025[c]	p=0.947 q=0.980
Visuospatial function	Pentagons Copy (MMSE)			0.88±0.326	0.84±0.369	p=0.460 q=0.918	0.88±0.326	0.84±0.369	p=0.460 q=0.918
Memory	Wechsler Memory Scale-III	Logical Memory	Immediate	28.39±12.708	29.71±13.125	p=0.386 q=0.918	28.39±12.708	29.71±13.125	p=0.386 q=0.918
			Delayed	16.02±10.380	16.68±10.061	p=0.620 q=0.918	16.02±10.380	16.68±10.061	p=0.620 q=0.918
		Visual Reproduction	Immediate	67.53±17.983[b]	66.73±19.181[b]	p=0.737 q=0.918	67.53±17.983[b]	66.73±19.181[b]	p=0.737 q=0.918
			Delayed	41.78±24.313[b]	42.66±24.475[b]	p=0.796 q=0.918	41.78±24.313[b]	42.66±24.475[b]	p=0.796 q=0.918
Psychomotor speed	Color Trails Test 1			65.22±33.858[b]	67.09±32.247[c]	p=0.717 q=0.918	65.22±33.858[b]	67.09±32.247[c]	p=0.717 q=0.918
Attention	Paced Auditory Serial Addition Test			64.31±18.085[d]	65.93±18.556[e]	p=0.727 q=0.918	64.31±18.085[d]	65.93±18.556[e]	p=0.727 q=0.918
Language	Language (MMSE)			4.92±0.281	4.83±0.463	p=0.261 q=0.918	4.92±0.281	4.83±0.463	p=0.261 q=0.918

The q-value used here is the False Discovery Rate (FDR), employed to correct the results of multiple comparisons involving 5 SNPs.

[b]Four missing value. [c]Thirteen missing value. [d]Twenty seven missing value. [e]Fifty eight missing value.

Supplementary Table 7. Moderation between CD33 SNPs and disease entity on cognitive functions

				rs12985029	rs3826656	rs33978622
				Gene-diagnosis interaction<br>(Disease-specific SNP effect)	Gene-diagnosis interaction<br>(Disease-specific SNP effect)	Gene-diagnosis interaction<br>(Disease-specific SNP effect)
Global cognitive screening	Mini-Mental State Examination			p=0.751<br>q=0.803	p=0.403<br>q=0.562	p=0.673<br>q=0.737
Executive function	Color Trails Test 2			p=0.505<br>q=0.618	p=0.179<br>q=0.360	p=0.229<br>q=0.376
Visuospatial function	Pentagons Copy (MMSE)			p=0.620<br>q=0.696	p=0.223<br>q=0.376	p=0.386<br>q=0.555
Memory	Wechsler Memory Scale-III	Logical Memory	Immediate	p=0.004<br>**q=0.037***<br>(HC: p=0.196;q=0.261<br>PD: p=0.584;q=0.584<br>HBV: p=0.045;q=0.090<br>HCV: p=0.007;**q=0.028***)	p<0.001<br>**q=0.007***<br>(HC: p=0.180;q=0.180<br>PD :p=0.095;q=0.127<br>HBV: p=0.025;**q=0.050***<br>HCV: p=0.002 ;**q=0.008***)	p=0.128<br>q=0.335
			Delayed	p=0.008<br>q=0.053<br>(HC: p=0.264;q=0.352<br>PD: p=0.524;q=0.524<br>HBV: p=0.034;q=0.068<br>HCV: p=0.017 ;q=0.068)	p<0.001<br>**q=0.007***<br>(HC: p=0.157;q=0.209<br>PD: p=0.211;q=0.211<br>HBV: p=0.069;q=0.138<br>HCV: p=0.001;**q=0.004***)	p=0.149<br>q=0.346
		Visual Reproduction	Immediate	p=0.214<br>q=0.376	p=0.131<br>q=0.335	p=0.489<br>q=0.618
			Delayed	p=0.084<br>q=0.276	p=0.072<br>q=0.255	p=0.158<br>q=0.346
Psychomotor speed	Color Trails Test 1			p=0.964<br>q=0.977	p=0.915<br>q=0.957	p=0.121<br>q=0.335
Attention	Paced Auditory Serial Addition Test			p=0.510<br>q=0.618	p=0.313<br>q=0.464	p=0.294<br>q=0.451
Language	Language (MMSE)			p=0.519<br>q=0.618	p=0.977<br>q=0.977	p=0.424<br>q=0.574

The q-value used here is the False Discovery Rate (FDR), employed to correct the results of multiple comparisons involving 3 SNPs. * *q<0.05*

Supplementary Table 8A. Moderation between CD33 SNPs and FIB4 on cognitive functions in individuals with chronic viral hepatitis

Genotypes				rs12985029		rs3826656		rs33978622	
Study Group (HBV+HCV)				n=154					
Age (years)				61.73±7.254					
Male/Female				77/77					
Education (years)				11.71±3.692					
Moderation analysis				Gene effect	Gene-inflammatory interaction	Gene effect	Gene-inflammatory interaction	Gene effect	Gene-inflammatory interaction
Global cognitive screening	Mini-Mental State Examination			p=0.804<br>q=0.945	p=0.301<br>q=0.860	p=0.295<br>q=0.860	p=0.092<br>q=0.613	p=0.747<br>q=0.945	p=0.485<br>q=0.945
Executive function	Color Trails Test 2			p=0.269<br>q=0.860	p=0.829<br>q=0.945	p=0.153<br>q=0.720	p=0.706<br>q=0.945	p=0.669<br>q=0.945	p=0.313<br>q=0.860
Visuospatial function	Pentagons Copy (MMSE)			p=0.669<br>q=0.945	p=0.749<br>q=0.945	p=0.856<br>q=0.945	p=0.608<br>q=0.945	p=0.344<br>q=0.860	p=0.679<br>q=0.945
Memory	Wechsler Memory Scale-III	Logical Memory	Immediate	p=0.156<br>q=0.720	p=0.884<br>q=0.945	p=0.317<br>q=0.860	p=0.446<br>q=0.945	p=0.213<br>q=0.860	p=0.749<br>q=0.945
			Delayed	p=0.022<br>q=0.264	p=0.394<br>q=0.914	p=0.038<br>q=0.285	p=0.786<br>q=0.945	p=0.029<br>q=0.285	p=0.250<br>q=0.860
		Visual Reproduction	Immediate	p=0.977<br>q=0.977	p=0.124<br>q=0.720	p=0.344<br>q=0.860	p=0.751<br>q=0.945	p=0.898<br>q=0.945	p=0.396<br>q=0.914
			Delayed	p=0.140<br>q=0.720	p=0.968<br>q=0.977	p=0.281<br>q=0.860	p=0.838<br>q=0.945	p=0.667<br>q=0.945	p=0.667<br>q=0.945
Psychomotor speed	Color Trails Test 1			p=0.001<br>**q=0.030***	p=0.001<br>**q=0.030***	p=0.011<br>q=0.220	p=0.021<br>q=0.264	p=0.333<br>q=0.860	p=0.037<br>q=0.285
Attention	Paced Auditory Serial Addition Test			p=0.658<br>q=0.945	p=0.833<br>q=0.945	p=0.518<br>q=0.945	p=0.871<br>q=0.945	p=0.598<br>q=0.945	p=0.865<br>q=0.945
Language	Language (MMSE)			p=0.658<br>q=0.945	p=0.965<br>q=0.977	p=0.582<br>q=0.945	p=0.503<br>q=0.945	p=0.807<br>q=0.945	p=0.826<br>q=0.945

The q-value used here is the False Discovery Rate (FDR), employed to correct the results of multiple comparisons involving 3 SNPs. * *q<0.05.*

Supplementary Table 8B: Moderation between FIB4 and CD33 SNPs (rs3865444 and rs12459419) on cognitive functions in individuals with chronic viral hepatitis

Genotypes				rs3865444		rs12459419	
Study Group (HBV+HCV)				n=153		n=154	
Age (years)				61.79±7.243		61.73±7.254	
Male/Female				77/76		77/77	
Education (years)				11.69±3.700		11.71±3.692	
Moderation analysis				Gene effect	Gene-inflammatory interaction	Gene effect	Gene-inflammatory interaction
Global cognitive screening	Mini-Mental State Examination			p=0.140<br>q=0.413	p=0.046<br>q=0.046	p=0.624<br>q=0.802	p=0.200<br>q=0.868
Executive function	Color Trails Test 2			p=0.132<br>q=0.413	p=0.704<br>q=0.972	p=0.136<br>q=0.413	p=0.625<br>q=0.972
Visuospatial function	Pentagons Copy (MMSE)			p=0.355<br>q=0.739	p=0.332<br>q=0.972	p=0.553<br>q=0.791	p=0.908<br>q=0.972
Memory	Wechsler Memory Scale-III	Logical Memory	Immediate	p=0.348<br>q=0.739	p=0.273<br>q=0.972	p=0.629<br>q=0.802	p=0.208<br>q=0.868
			Delayed	p=0.075<br>q=0.404	p=0.756<br>q=0.972	p=0.071<br>q=0.404	p=0.922<br>q=0.972
		Visual Reproduction	Immediate	p=0.845<br>q=0.952	p=0.187<br>q=0.868	p=0.953<br>q=0.984	p=0.190<br>q=0.868
			Delayed	p=0.203<br>q=0.507	p=0.933<br>q=0.972	p=0.414<br>q=0.786	p=0.771<br>q=0.972
Psychomotor speed	Color Trails Test 1			p=0.003<br>**q=0.049***	p=0.002<br>**q=0.027***	p=0.001<br>**q=0.038***	P<0.001<br>**q=0.015***
Attention	Paced Auditory Serial Addition Test			p=0.132<br>q=0.413	p=0.704<br>q=0.972	p=0.965<br>q=0.984	p=0.418<br>q=0.972
Language	Language (MMSE)			p=0.984<br>q=0.984	p=0.692<br>q=0.972	p=0.768<br>q=0.914	p=0.566<br>q=0.972

The q-value used here is the False Discovery Rate (FDR), employed to correct the results of multiple comparisons involving 5 SNPs. * *q<0.05*.

Supplementary Table 9. Moderation between CD33 SNPs and FIB4 on cognitive functions in individuals with chronic viral hepatitis with the addition of AST and ALT as covariates

Genotypes				rs12985029		rs3826656		rs33978622	
Study Group (HBV+HCV)				n=154					
Age (years)				61.73±7.254					
Male/Female				77/77					
Education (years)				11.71±3.692					
Moderation analysis				Gene effect	Gene-inflammatory interaction	Gene effect	Gene-inflammatory interaction	Gene effect	Gene-inflammatory interaction
Global cognitive screening	Mini-Mental State Examination			p=0.952<br>q=0.990	p=0.445<br>q=0.998	p=0.683<br>q=0.838	p=0.351<br>q=0.998	p=0.893<br>q=0.990	p=0.744<br>q=0.998
Executive function	Color Trails Test 2			p=0.314<br>q=0.648	p=0.808<br>q=0.998	p=0.099<br>q=0.314	p=0.513<br>q=0.998	p=0.607<br>q=0.828	p=0.402<br>q=0.998
Visuospatial function	Pentagons Copy (MMSE)			p=0.698<br>q=0.838	p=0.952<br>q=0.998	p=0.957<br>q=0.990	p=0.702<br>q=0.998	p=0.523<br>q=0.785	p=0.838<br>q=0.998
Memory	Wechsler Memory Scale-III	Logical Memory	Immediate	p=0.056<br>q=0.280	p=0.856<br>q=0.998	p=0.083<br>q=0.311	p=0.917<br>q=0.998	p=0.105<br>q=0.314	p=0.421<br>q=0.998
			Delayed	p=0.007<br>q=0.053	p=0.336<br>q=0.998	p=0.006<br>q=0.053	p=0.653<br>q=0.998	p=0.014<br>q=0.084	p=0.155<br>q=0.998
		Visual Reproduction	Immediate	p=0.867<br>q=0.990	p=0.190<br>q=0.998	p=0.158<br>q=0.395	p=0.781<br>q=0.998	p=0.324<br>q=0.648	p=0.975<br>q=0.998
			Delayed	p=0.115<br>q=0.314	p=0.865<br>q=0.998	p=0.070<br>q=0.300	p=0.375<br>q=0.998	p=0.418<br>q=0.697	p=0.998<br>q=0.998
Psychomotor speed	Color Trails Test 1			p=0.002<br>q=0.053	P<0.001<br>**q=0.021***	p=0.006<br>q=0.053	p=0.011<br>q=0.165	p=0.314<br>q=0.648	p=0.023<br>q=0.230
Attention	Paced Auditory Serial Addition Test			p=0.550<br>q=0.786	p=0.981<br>q=0.998	p=0.357<br>q=0.670	p=0.677<br>q=0.998	p=0.385<br>q=0.679	p=0.776<br>q=0.998
Language	Language (MMSE)			p=0.640<br>q=0.835	p=0.810<br>q=0.998	p=0.465<br>q=0.734	p=0.289<br>q=0.998	p=0.996<br>q=0.996	p=0.657<br>q=0.998

The q-value used here is the False Discovery Rate (FDR), employed to correct the results of multiple comparisons involving 3 SNPs. * *q<0.05.*
